# Physics‐Informed Emulation of Systemic Circulation for Fast Parameter Estimation and Uncertainty Quantification

**DOI:** 10.1002/cnm.70147

**Published:** 2026-02-13

**Authors:** William Ryan, Alyssa Taylor‐LaPole, Mette Olufsen, Dirk Husmeier, Vladislav Vyshemirskiy

**Affiliations:** ^1^ School of Mathematics and Statistics, University of Glasgow Glasgow UK; ^2^ Rice University Houston Texas USA; ^3^ North Carolina State University Raleigh North Carolina USA

**Keywords:** computational fluid dynamics, FALD, Fontan, perfusion, wall shear stress

## Abstract

There are many computational models set up to predict blood flow and pressure in vascular networks. Methods for a single forward solution of such models are well established but become problematic in clinical applications, where model calibration and patient‐specific parameter estimation call for repeated forward simulations of the model requiring substantial computational costs. A potential workaround is emulation, which approximates the original mathematical model by a statistical or machine learning surrogate model. Our methodological framework is based on physics‐informed neural networks, with a particular focus on patient‐specific model calibration. Once fully trained, our machine learning model predicts flow and pressure waveforms in a fraction of the time required by the numerical solver, enabling fast parameter inference and inverse uncertainty quantification. The proposed framework is applied to clinical data from four patients diagnosed with a Double Outlet Right Ventricle (DORV), a congenital heart defect where both the aorta and main pulmonary artery connect to the right ventricle, potentially leading to insufficient oxygen delivery to the body and hence requiring careful blood flow monitoring. We assess the performance of our method in a comparative evaluation study that includes several alternative state‐of‐the‐art machine learning methods, and we quantify the improvement achieved in terms of accuracy and efficiency gains.

## Graphical abstract

This work presents an innovative approach to modelling 1D fluid dynamics in complex networks using physics‐informed neural networks as surrogate models. By integrating physics‐based constraints with data‐driven learning, we develop an efficient and generalisable framework for uncertainty quantification and parameter estimation in real‐world applications.

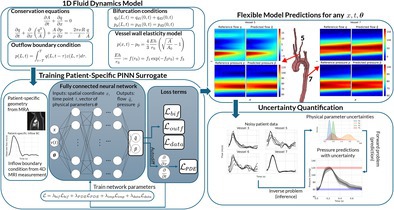

## 1 Introduction

One‐dimensional (1D) computational fluid dynamics (CFD) models have long been used for simulating haemodynamics in vascular networks including studies by Reymond et al. [1], Mariscal‐Harana et al. [2], Johnson et al. [3], and Olufsen et al. [4]. They represent key haemodynamic properties such as pressure, flow rate, wave propagation, and shear stress, making them relevant to clinical decision‐making. However, while the computational efficiency gain over 3D models enables forward simulations for fixed model parameters in real time [5], 1D CFD models are computationally too complex for real‐time model calibration and parameter estimation (the so‐called *inverse problem*). This would require a substantial number of forward simulations with different model parameters as part of an iterative optimisation or sampling scheme. Such patient‐specific model calibration and physical parameter inference are of critical importance for clinical applications, for instance, as part of a clinical decision support system based on proper inverse uncertainty quantification (IUQ).

Surrogate models can be introduced to approximate the outputs of 1D fluid dynamics simulations, resulting in a significant reduction in computation time. After incurring an initial time cost in the form of a training period, such models can make rapid predictions. This allows for extensive simulations in terms of performing IUQ for potential surgical outcomes, such as the construction of a Fontan circuit in single‐ventricle infants [6, 7]. Applications of data‐driven surrogate models to CFD (in general) and cardiovascular modelling (in particular) have been extensively researched, for example, using polynomial chaos expansions (PCE) [8, 9], Gaussian Process (GP) regression [10], and deep learning [11, 12]. A disadvantage of these purely data‐driven surrogate models is that they may not fully capture underlying dynamics. As a result, their performance may suffer when extrapolating to conditions outside the training domain. This shortcoming can be addressed by explicitly integrating physics‐based information into the surrogate modelling process.

One of the first approaches to modelling fluid dynamics using physics‐informed machine learning was introduced by Raissi et al. [13], where 2D and 3D Navier–Stokes problems were tackled. In this study, the networks were trained using (synthetic) observational data and governing equations where Reynolds and Peclet numbers were inferred as part of the training process. Their work included an application to cardiovascular fluid dynamics, namely in a 2D stenosis toy problem. In a similar vein, Garay et al. [14] used physics‐informed neural networks to infer Windkessel parameters, again as part of the network optimisation. Their study used 2D MRI flow and pressure data observed at vessel midpoints over one period to inform these parameters, which defined the model's boundary condition. Of particular interest is the work carried out by Kissas et al. [15], where PINNs were applied to a 1D model of blood flow in both synthetic and patient‐specific vessels. The authors utilised observational flow and cross‐sectional area data at vessel midpoints to train a physics‐informed neural network, which could then be used to predict blood pressure, flow, and vessel cross‐sectional area at any time point or location in their respective domains, with a particular focus on non‐invasive aortic pressure predictions. The applications ranged from a three vessel system including one bifurcation with synthetic data to a seven vessel network with three bifurcations applied to real patient data. Windkessel parameters were inferred post‐training using predictions of flow and pressure at the ends of the outflow vessels. Yang et al. [16] introduced a general class of Bayesian physics‐informed neural networks (b‐PINNs) designed to handle noisy observational data. By modelling uncertainty in the network weights, their approach yielded predictive intervals rather than a single deterministic solution. The authors employed both Markov Chain Monte Carlo (MCMC) and variational inference (VI) methods to infer the network parameters. The methodology was tested on a variety of 1D and 2D PDEs toy problems. Sun and Wang [17] approached the problem of modelling 2D fluid flow from noisy or sparse data by using b‐PINNs and a slightly modified VI method. Their application included a toy problem of an idealised stenosis. They demonstrated that hybrid data‐driven and physics‐informed models (i.e., models that utilise both observational (simulation) data and PDE residuals) learn solutions faster and more accurately than purely PDE‐residual‐based learning methods. More recently, Huang et al. [18] tackled a one‐fluid cavitation model problem using a joint data‐driven and physics‐informed approach, finding that combining the two sources of information led to more accurate results than previously achieved. Note that in this case, data‐driven means using simulation data obtained by numerically solving the equations. Investigations into fully data‐free (i.e., fully physics‐informed) modelling have also been carried out. Sun et al. [19] introduced a surrogate model for steady‐state 2D flow, which did not require data to fit, as long as boundary conditions were imposed correctly. The work focused on a toy problem of idealised stenotic flow. This approach included an input parameter ν in the model, in addition to the spatial coordinates, which corresponded to fluid viscosity.

Isaev et al. [20] modelled flow in a steady‐state system of four vessels resulting from the Fontan procedure in a data‐driven manner to investigate how perfusion changed given a change in geometry. Their model accepted geometry parameters (vessel radii and bifurcation angles) and boundary conditions (pressure values at the inlet and outlets) as inputs and outputted flow predictions at single points in each of the four vessels. Physics‐informed machine learning continues to be a heavy focus in both the machine learning literature (sampling strategies [21, 22], avoiding failure modes [23], architectures [24]) and applied engineering problems [25, 26, 27].

When making a model patient‐specific for medical applications, each parameter adaptation calls for another numerical integration of the PDEs. The accumulated time can reach days or weeks. However, since predictions made using a trained machine‐learning model can be done in a fraction of the time required by a standard numerical solver, the overall computational costs of Bayesian inference can be reduced by several orders of magnitude to time frames acceptable for clinical decision making. The focus of the present work is on implementing physics‐informed machine learning for emulation in the context of Bayesian inference and inverse uncertainty quantification to understand aortic remodelling in infants with a Fontan circulation. The novelty lies in a substantial upscaling of the model complexity to a 1D fluid dynamics model of a large haemodynamic network of up to 17 interconnected blood vessels. In addition to predicting flow and pressure as a function of space and time, our model accepts biophysical parameters as inputs. These parameters define the elasticity of the vessel walls in the large and small vessels, as well as the geometry of the network of small vessels. Given noisy, clinical flow waveforms extracted from 4D‐magnetic resonance images (MRIs), our aim is to carry out Bayesian inference and inverse uncertainty quantification of the physical parameters using posterior sampling. To this end, we need model outputs for repeatedly varying parameters as part of an established sampling routine, such as Hamiltonian Monte Carlo [28] (HMC).

**FIGURE 1:**
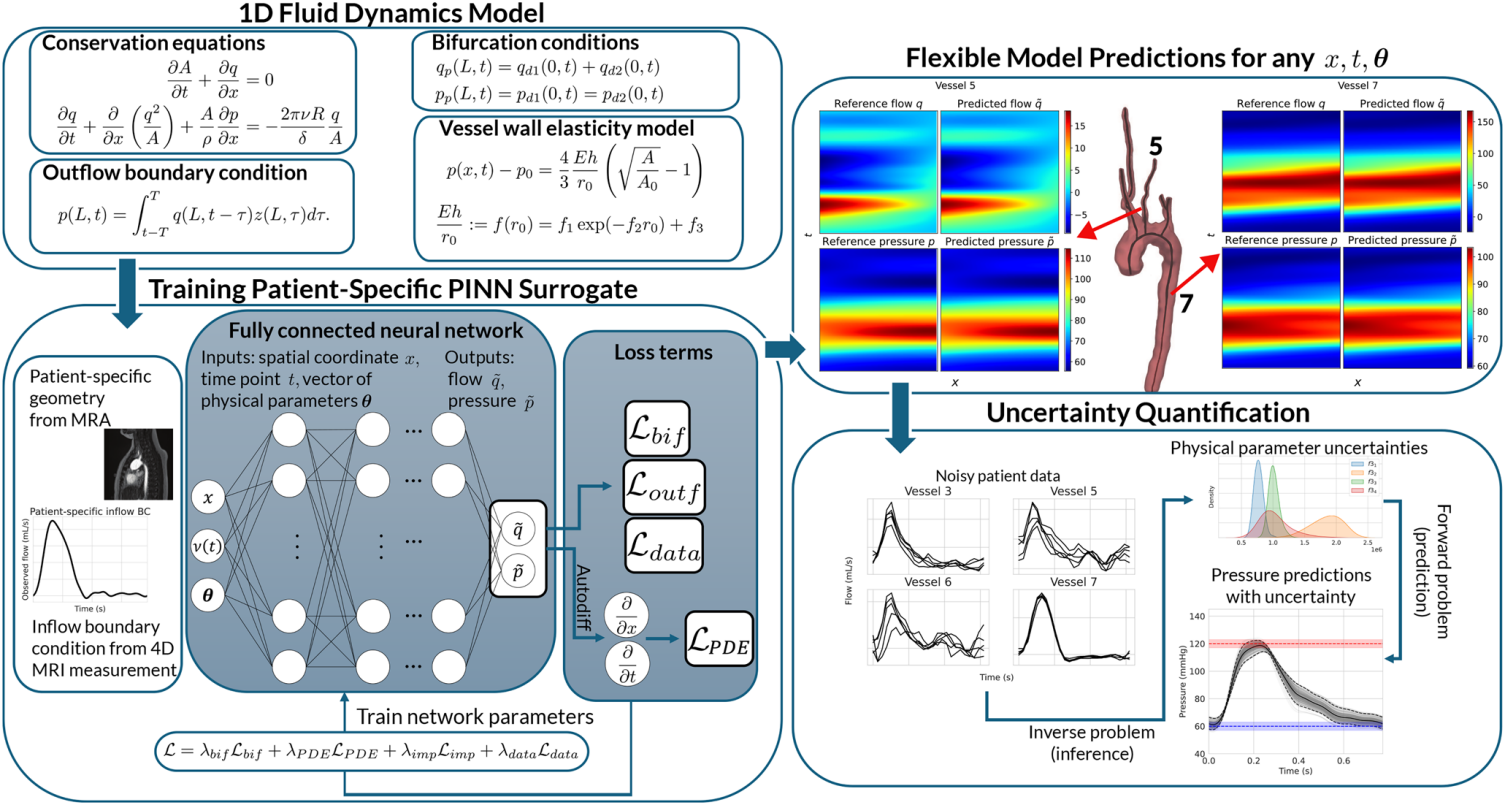
Overview of the modelling and inference pipeline. The process begins with the 1D fluid dynamics model (top‐left panel) and patient‐specific geometry extracted from MRA data. A PINN is then trained as a surrogate model using both clinical inflow boundary conditions from 4D MRI and embedded physical constraints such as conservation laws and boundary conditions (bottom‐left panel). Once trained, the PINN is used to perform fast forward predictions of flow and pressure at any coordinate in space, time and physical parameter space (top‐right panel) and inverse inference of biophysical parameters, enabling uncertainty quantification and non‐invasive pressure predictions (bottom‐right panel). The top‐right panel displays flow and pressure fields predicted by the trained PINN model alongside fields obtained via a high‐fidelity PDE solver in two different vessels.

This study is organised as follows: Section 2 introduces the multiscale 1D fluid dynamics model and Section 3 the physics‐informed neural networks used as surrogate models. The implementation workflow is presented in Figure 1. Numerical results are reported in Section 4, demonstrating predictive performance compared to proven surrogate models and the ability to perform uncertainty quantification in two arterial network models: a proof‐of‐concept application in a smaller network consisting of 9 vessels (the aorta and those immediately branching off), as well as a more realistic model composed of 17 vessels (extension of the 9 vessel model including head and neck vessels) with accompanying real patient data. Finally, findings are summarised in Section 5.

## 2 Fluid Dynamics Model and Data

### 2.1 Vascular Network and Haemodynamic Data

The aorta, head, and neck vessel geometry is taken from Taylor‐LaPole et al. [29] and Paun et al. [30]. Vascular dimensions are determined by first segmenting the vasculature from patient MRA images, creating a 3D‐rendered volume of the vessels. Then, the centerlines are generated throughout each vessel. From the centerlines, a directed labelled tree is defined including vessel connectivity, length, and radii.

**FIGURE 2:**
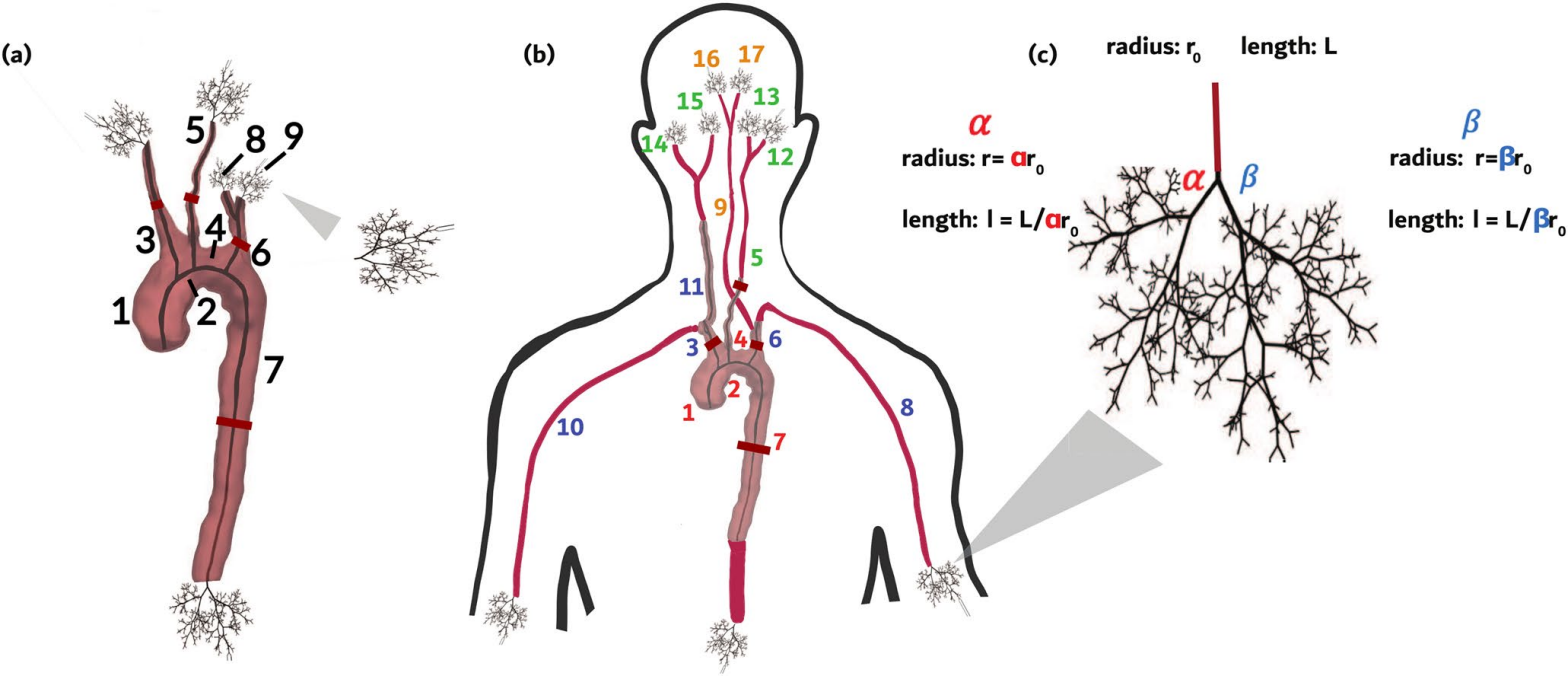
Diagrams of the two models considered in this work. (a) Layout of the arteries in the 9‐vessel model. Structured trees, which describe the outflow boundary condition, are attached to the terminal vessels. (b) Diagram depicting the 17‐vessel network. The numbers corresponding to each vessel are coloured by group, where each group is assigned its own vessel stiffness value. (c) The terminal vessels are indicated by ending in a structured tree of small vessels that determines the outflow boundary conditions. The red lines intersecting Vessels 3, 5, 6 and 7 in both (a) and (b) indicate the approximate location at which flow data was available to infer physical parameters with.

Flow and cuff pressure data are collected from four double outlet right ventricle (DORV) patients. These patients are born with the pulmonary artery and the aorta connected to the functioning right ventricle. To prevent the mixing of systemic (oxygenated) and pulmonary (deoxygenated) blood flow, the DORV patients included in this study have previously undergone surgeries to obtain a single‐ventricle Fontan circulation, that is, the pulmonary arteries are detached from the right ventricle and connected to the inferior and superior vena cava. The flow waveforms are extracted from 4D MRIs [29, 31, 32], and these 4D‐MRI images are then registered on the MRA images used to segment the networks, as described in [29, 31, 32]. The time‐resolved 4D flow MRI data in the ascending aorta is used as the inflow boundary condition for the model. Flow data is also determined in the innominate artery, left common carotid, left subclavian artery, and descending aorta (Vessels 3, 5, 6 and 7 in Figure 2b). Finally, diastolic and systolic pressure measurements were obtained via cuff measurements in the supine position with a sphygmomanometer.

### 2.2 One‐Dimensional (1D) Arterial Model With Structured Tree Boundary Conditions

The 1D model integrates the axisymmetric Navier Stokes equations for an incompressible fluid to predict periodic blood flow, pressure, and cross‐sectional area in each vessel as a function of time. Each vessel is assumed to be cylindrical with a thin, impermeable wall. We assume that the flow is Newtonian, incompressible, and axisymmetric.

The computational domain is represented by a network of interconnected vessels, and has two parts: large vessels and small vessels. The connectivity and geometry (length and radius) of the large vessels can be extracted from images, while a self‐similar structured tree represents the small vessel network. In the large vessels, blood flow and pressure are predicted by solving the non‐linear 1D equations, whereas in the small vessels, we solve a linear 1D model. From the perspective of the large vessels, haemodynamic predictions in the small vessels form the boundary conditions. We prescribe a flow waveform at the inlet to the network, and assume that flow is conserved and pressure is continuous at each junction (both in the large and small networks). The non‐linear system of PDEs in the large vessels is hyperbolic, that is, for each vessel, we require a boundary condition at each end. The equations are non‐dimensionalised and solved using an in‐house, two‐step Lax‐Wendroff solver [33]. This algorithm is suitable for studying blood flow in arteries, where we do not anticipate shock under physiological flow conditions.

#### 2.2.1 Networks—Computational Domain

##### 2.2.1.1 Large Vessel Networks

The present study examines haemodynamics in two vascular networks. Network 1 (Figure 2a) includes 9 large vessels: the ascending aorta, the aortic arch, the descending aorta, and vessels branching into the neck. The length l (cm) and unstressed vessel radii $r_{0}$ (cm) of each vessel are obtained from the skeletonised network data. It is important to note that this smaller network includes only those vessels that are visible and reliably segmented in the medical images. Network 2 (Figure 2b) extends network 1 to incorporate vessels outside of the imaged region. This larger network includes 17 large vessels, with vessels extending into the arms and brain. Note all junctions, in both the large and small networks, are bifurcations, that is, a given vessel will always have exactly two daughter vessels. Detailed descriptions of these networks are provided in the modelling study by Taylor‐LaPole et al. [29] which was followed closely, besides from applying a stronger tapering effect to the descending aorta.

##### 2.2.1.2 Small Vessel Networks

The small vessel networks (Figure 2c) (attached at the terminal vessels of the large vessels, Figure 2a,b) consist of self‐similar bifurcating trees in which the unstressed vessel radii $r_{0}$ (cm) of the daughter vessels are scaled relative to the parent vessel as

(1) $${\left(r_{0}\right)}_{d1}=\alpha {\left(r_{0}\right)}_{p},\;{\left(r_{0}\right)}_{p}=\beta {\left(r_{0}\right)}_{d2}$$

The scaling factors are chosen such that the vessel labelled daughter 1 (d1) is always bigger than daughter 2 (d2). We assume that the radii of the daughter vessels are smaller than those of the parent vessel (0<α<β<1). The structured tree continues to bifurcate until the radii are smaller than a set minimum radius $r_{\min}$. Self‐similarity is also reflected in the length of the vessel, which is set for each vessel at $L=l_{\mathrm{rr}}\cdot r_{0}$, where $l_{\mathrm{rr}}$ is the length‐to‐radius ratio.

#### 2.2.2 Large Vessel Equations

The system of equations solved in each large vessel relates volumetric flow $q\left(x,t\right)$ (mL/s), pressure $p\left(x,t\right)$ (mmHg), and cross‐sectional area $A\left(x,t\right)=\mathrm{\pi R}{\left(x,t\right)}^{2}$ (cm^2^). For each cardiac cycle, we ensure that 0<t<T and $x\in \left(0,L\right)$, where L is the length of the vessel. The system of equations satisfying conservation of mass and balance of momentum is given by

(2) $$\frac{\partial A}{\partial t}+\frac{\partial q}{\partial x}=0,$$

(3) $$\frac{\partial q}{\partial t}+\frac{\partial }{\partial x}\left(\frac{q^{2}}{A}\right)+\frac{A}{\rho }\frac{\partial p}{\partial x}=-\frac{2\mathrm{\pi \nu R}}{\delta }\frac{q}{A},$$

where ρ=1.057 (g/cm^3^) is density, μ=0.032 (g/cm/s) is viscosity and $\nu =\frac{\mu }{\rho }$ (cm^2^/s) denotes the kinematic viscosity. In the present study, we kept these quantities constant with values obtained from literature [34], as they were not expected to change much from patient to patient. The equations are derived by assuming a Stokes boundary layer with thickness $\delta =\sqrt{\mathrm{\nu T}/2\pi }$.

To close the system of equations, a linear stress–strain model relating pressure to area is imposed.

(4) $$p\left(x,t\right)-p_{0}=\frac{4}{3}\frac{\mathrm{Eh}}{r_{0}}\left(\sqrt{\frac{A}{A_{0}}}-1\right),$$

(5) $$\frac{\mathrm{Eh}}{r_{0}}=f\left(r_{0}\right)=f_{1}\exp\left(-f_{2}r_{0}\right)+f_{3},$$

where $p_{0}$ (mmHg) is the unstressed pressure and $r_{0}$ (cm), $A_{0}=\pi r_{0}^{2}$ (cm^2^) are the equivalent unstressed radius and cross‐sectional area, respectively. The latter are obtained from the segmentation of vascular networks. To account for the increased stiffening with the decrease in vessel radius, Young's modulus E (g/cm/s^2^) and wall thickness h (cm) are related to the reference vessel radius $r_{0}$ as an exponentially tapering function $f\left(r_{0}\right)$ parametrised by three parameters $f_{1}$, $f_{2}$ and $f_{3}$.

#### 2.2.3 Boundary Conditions

From the perspective of the large networks (shown in Figure 2a,b), three types of boundary conditions are imposed: at the inlet, at each junction, and the terminal vessels.

##### 2.2.3.1 Inflow

At the network inlet, we impose a smooth periodic flow waveform $q\left(0,t\right)$ (mL/s) obtained by generating a smooth waveform from the measured flow in the ascending aorta, discretised at the number of time‐points required by the numerical solver.

##### 2.2.3.2 Junctions (Bifurcations)

At the junctions, we impose conservation of flow and continuity of pressure, that is, the flow and pressure at the end of each parent vessel (subscript p) are related to the values at the inlet of the two daughter vessels (subscript d1 and d2). These conditions give

(6) $$q_{p}\left(L,t\right)=q_{d1}\left(0,t\right)+q_{d2}\left(0,t\right)$$

(7) $$p_{p}\left(L,t\right)=p_{d1}\left(0,t\right)=p_{d2}\left(0,t\right).$$

##### 2.2.3.3 Outflow Conditions

At the end of the terminal vessels (within the tree of large vessels), we impose an outlet condition via an impedance obtained by solving a system of linearised equations in the structured tree model described in detail in [4].

Within each vessel, the linearised equations can be solved analytically in the frequency domain, generating expressions for impedance $Z\left(x,\omega \right)=P\left(x,\omega \right)/Q\left(x,\omega \right)$. To generate a solution in the tree, we determine the impedance at the beginning of each vessel x=0 as a function of the impedance at the end of the vessel x=L as

(8) $$Z\left(0,\omega \right)=\frac{{\mathrm{ig}}_{\omega }^{-1}\sin\left(\mathrm{\omega L}/c\right)+Z\left(L,\omega \right)\cos\left(\mathrm{\omega L}/c\right)}{\cos\left(\mathrm{\omega L}/c\right)+{\mathrm{ig}}_{\omega }Z\left(L,\omega \right)\sin\left(\mathrm{\omega L}/c\right)},\;\omega \neq 0$$

(9) $$Z\left(0,\omega \right)=\frac{8\mu l_{\mathrm{rr}}}{\pi r_{0}^{3}}+Z\left(L,\omega \right),$$

where $g=\sqrt{{\mathrm{CA}}_{0}\left(1-F_{J}\right)/\rho }$ (n.d.) and the wave speed $c=\sqrt{\frac{A_{0}\left(1-F_{J}\right)}{\mathrm{\rho C}}}$ (n.d.). In these equations $A_{0}$ is the unstressed vessel radius, $C=\frac{\partial A}{\partial p}=\frac{3A_{0}r_{0}}{2\mathrm{Eh}}$ is the vessel compliance, and $F_{J}=\frac{2J_{1}\left(w_{0}\right)}{w_{0}J_{0}\left(w_{0}\right)},w_{0}=i^{3}r_{0}^{2}\omega /\nu$ is a ratio of Bessel functions $J_{i},i=0,1$ (functions of the Womersley number $w_{0}$) arising from the solution of the linearised PDE [4]. Similar to the large vessel network, the equation in the interior is combined with junction conditions, which in the frequency domain are given by

(10) $$\frac{1}{Z_{p}\left(L,\omega \right)}=\frac{1}{Z_{d1}\left(0,\omega \right)}+\frac{1}{Z_{d2}\left(0,\omega \right)}.$$

Using a recursive algorithm (described in detail in [4]), the solution is propagated to the root of the structured tree, where the impedance defines the relationship between flow and pressure as follows:

(11) $$p\left(L,t\right)=\int_{t-T}^{T}q\left(L,t-\tau \right)z\left(L,\tau \right)\mathrm{d\tau},$$

where *z* (*x*, *t*) is obtained by an inverse periodic Fourier transform of $Z\left(x,\omega \right)$. Alternatively, by the convolution theorem,

(12) $$ℱ\left(p\left(L,t\right)\right)=ℱ\left(q\left(L,t\right)\right)ℱ\left(z\left(L,\tau \right)\right),$$

where ℱ denotes the Fourier transform.

#### 2.2.4 Model Parameters

The fluid dynamics model has three types of parameters specifying: *the computational domains* (the large and small vessel networks), *fluid properties*, and *physiological properties*. Nominal values and a priori bounds for all parameters, as well as a subset of identifiable parameters via a sensitivity analysis, are determined from data and results reported in previous studies [29, 30]. We examine haemodynamics in two networks (Figure 2a,b) with N=9 and N=17 large vessels, respectively. For the N=9 vessel network, the vessel parameters are the same for all vessels, while for the N=17 network, we include four groups denoting vessels with varying properties (see Figure 2b for the groupings). Below we summarise all model parameters noting identifiable parameters for each type.

- *Large vessel networks:* Large vessel parameters include estimates of vessel radii ${\left(r_{0}\right)}_{i},i=1,\ldots ,N$, vessel length $l_{i},i=1,\ldots ,N$, and vessel connectivity. For both networks, estimates for these parameters are obtained from segmentation of the MRA images.
- *Small vessel networks:* Terminal vessels of the large vessel networks are connected to structured trees characterised by four parameters: α, β, $l_{\mathrm{rr}}$, and $r_{\min}$. The parameter $r_{\min}$ specifying the size of the smallest vessels is kept fixed. For the terminal tree attached to the large descending aorta $r_{\min}=0.001$ and for the structured tree attached to the descending aorta and for the terminal vessels in the head and neck $r_{\min}=0.0001$. The parameters $\left(\alpha ,\beta ,l_{\mathrm{rr}}\right)$ are correlated. Similar to the original study [29], α is inferred, while β and $l_{\mathrm{rr}}$ kept fixed at their nominal values.
- *Fluid properties* include blood density ρ and viscosity μ. Parameters associated with these quantities are fixed for all simulations (see above), as they are easy to measure in vivo and have been extensively reported in haemodynamics studies.
- *Biophysical parameters* include parameters denoting vessel compliance, that is, parameters required to specify $\mathrm{Eh}/r_{0}$, that is, ($f_{i_{1}},f_{i_{2}},f_{i_{3}},\;i=s,l$). These parameters appear both in both the large (i=l) and small (i=s) networks. Again, the parameters of the large and small vessels are correlated. For both networks we fix $f_{1}$ for all large vessels and structured trees, we infer one value of $f_{i_{2}}$, i=l,s for the large and small vessels, respectively, and group specific values for $f_{i_{\left(3,j\right)}}$ (one value j=1 for the 9‐vessel network and 4 values j=1,…,4 for the 17‐vessel network).

In summary, for the 9‐vessel network (Figure 2a), we infer 5 parameters

(13) $$\theta =\left(f_{l_{2}},f_{s_{2}},f_{l_{3}},f_{s_{3}},\alpha \right)$$

and for the 17‐vessel network (Figure 2b), we infer 10 parameters

(14) $$\theta =\left(f_{l_{2}},f_{s_{2}},f_{l_{\left(3,j\right)}},f_{s_{\left(3,j\right)}},\alpha \right),\;j=1,\ldots ,4.$$

## 3 Methods

### 3.1 Training the Physics‐Informed Emulator

An independent, fully connected feed‐forward neural network, also known as a multilayer perceptron (MLP), is assigned separately to each vessel in the system. A comprehensive comparison of network architectures for blood flow modelling was carried out by Moser et al. [35], where it was found that fully connected feedforward networks provide a favourable balance between prediction accuracy and training time. The networks accept spatial location in the axial direction $x\in \left[0,L\right]$, where L (cm) is the vessel‐specific length, time point t, and biophysical parameters θ as inputs, and output flow and pressure values

(15) $$u_{\mathrm{NN}}:\left(x,t,\theta \right)\mapsto \left(\hat{q},\hat{p}\right).$$

Note that the domain of the spatial input x is different for each vessel, reflecting the vessel‐specific lengths. The parameters belonging to the vector of biophysical parameters θ are the quantities to be inferred and remain a modelling choice.

A neural network with L layers is defined as a composition of functions

(16) $$u_{\mathrm{NN}}\left(x,t,\theta \right)=\left(u_{L}\circ u_{L-1}\circ \cdots \circ u_{1}\right)\left(x,t,\theta \right).$$

Each layer $u_{\ell }:{\mathbb{R}}^{n_{\ell -1}}\to {\mathbb{R}}^{n_{\ell }}$ is given by

(17) $$u_{\ell }\left(z\right)=\sigma_{\ell }\left(W_{\ell }z+b_{\ell }\right),$$

where $W_{\ell }\in {\mathbb{R}}^{n_{\ell }\times n_{\ell -1}}$ is the weight matrix, $b_{\ell }\in {\mathbb{R}}^{n_{\ell }}$ is the bias vector, and $\sigma_{\ell }$ is a (typically non‐linear) activation function applied elementwise. The complete set of parameters is $\omega =\left\{W_{\ell ,m},b_{\ell ,m}|\ell =1,\ldots ,L,m=1,\ldots ,M\right\},$ where M denotes the total number of vessels in the system. Note that the cross‐sectional vessel area A is an additional quantity present in the equations of the 1D model and can be easily found by rearranging Equation (5) given a predicted pressure value and vessel stiffness parameters:

(18) $$\hat{A}\left(x,t,\theta \right)=A_{0}{\left(\frac{3}{4}\frac{r_{0}}{\mathrm{Eh}}\left(\hat{p}\left(x,t,\theta \right)-p_{0}\right)+1\right)}^{2}.$$

The 1D model outlined in Section 2.2 imposes several constraints that the flow and pressure solutions must adhere to. These form a collection of loss functions that must be satisfied for the neural network to provide the correct solution to the problem.

### 3.2 Exact Imposition of Inlet Boundary Condition and Periodicity

Solving the patient‐specific fluid dynamics problem requires a set of geometry parameters (lengths and radii of arteries) and an inflow profile. The inflow profile imposed at the inlet of the first vessel in the system remains fixed and periodic, given period T (s), irrespective of additional physiological input parameters. Furthermore, since the system is assumed to be periodic, flow and pressure should be equal at all times t=kT, k∈ℤ. Both of these conditions are imposed exactly as described in the subsequent sections, rather than being learnt through loss functions.

#### 3.2.1 Inflow

Embedding the inflow boundary condition into the solution requires a representation of flow as a continuous, at least one‐time differentiable function of time. When numerically solving the PDEs, the inflow exists as a vector of flow data on a sufficiently fine grid to ensure numerically stable solutions. Incorporating a deterministic inflow into the PINN solution requires a continuous inflow profile. GP [36] regression offers itself as an excellent choice for the following reasons: GPs are fast to apply and train given the number of points expected in this application (<100), satisfy the differentiability requirement given appropriate choice of covariance function (kernel) and periodicity can be enforced through a periodic kernel. In our study, a Matérn 5/2 kernel wrapped inside a periodic kernel [37] was chosen for modelling the inflow. This kernel is twice differentiable, which appears more realistic than the more common infinitely differentiable (and hence potentially over‐smooth) Squared Exponential (SE) kernel. Having fitted the GP and optimised hyperparameters via maximising the marginal likelihood, the posterior predictive mean was used as the surrogate inlet boundary condition.

Let $q_{\text{in}}\left(t\right)$ be the inflow profile over one period and $\hat{q}\left(x,t,\theta \right)$ the neural network's flow prediction of the first vessel in the system. The corresponding PINN can be augmented to satisfy the inflow profile exactly at location x=0 by mixing the two outputs [38].

(19) $$\tilde{q}\left(x,t,\theta \right)=\cos\left(\frac{\pi }{2}\frac{x}{L}\right)q_{\text{in}}\left(t\right)+\sin\left(\frac{\pi }{2}\frac{x}{L}\right)\hat{q}\left(x,t,\theta \right),$$

where L (cm) denotes the length of the vessel and $x\in \left[0,L\right]$.

#### 3.2.2 Periodicity

Satisfying one‐dimensional periodicity exactly requires imposing the condition

(20) $$u_{\mathrm{NN}}\left(x,t,\theta \right)=u_{\mathrm{NN}}\left(x,t+\mathrm{kT},\theta \right),k\in \mathbb{Z},$$

on the neural network, where T is the period of the system. A change of variable is introduced in the first layer of the neural network for the variable t, which naturally satisfies the constraint in Equation (20)

(21) $$v:t\mapsto \left(\cos\left(\frac{2\mathrm{\pi t}}{T}\right),\;\sin\left(\frac{2\mathrm{\pi t}}{T}\right)\right).$$

The input‐augmented neural network is then represented as follows:

(22) $${\tilde{u}}_{\mathrm{NN}}:\left(x,\nu \left(t\right),\theta \right)\mapsto \left(\tilde{q},\tilde{p}\right),$$

where $\tilde{q}$ is given by Equation (19) in the inflow vessel, and otherwise flow and pressure are the raw neural network outputs: $\tilde{q}=\hat{q},\tilde{p}=\hat{p}$.

### 3.3 Loss Functions

#### 3.3.1 Bifurcations

The outputs of the neural network representing flow and pressure in each vessel in the artery tree must satisfy the same continuity conditions as presented in Section 2.2. This is incorporated into the training process via a soft constraint, using mean‐squared error loss functions. Given a bifurcating parent vessel of length $L_{p}$ with 2 daughter vessels, for a time point t and vector of biophysical parameters θ, the residuals between flow predictions are defined as

(23) $$r_{q}\left(t,\theta \right)≔{\tilde{q}}_{p}\left(L_{p},t,\theta \right)-{\tilde{q}}_{d1}\left(0,t,\theta \right)-{\tilde{q}}_{d2}\left(0,t,\theta \right)$$

The pressure continuity residuals are split into two equations:

(24) $$r_{p1}\left(t,\theta \right)≔{\tilde{p}}_{p}\left(L_{p},t,\theta \right)-{\tilde{p}}_{d1}\left(0,t,\theta \right),$$

(25) $$r_{p2}\left(t,\theta \right)≔{\tilde{p}}_{p}\left(L_{p},t,\theta \right)-{\tilde{p}}_{d2}\left(0,t,\theta \right).$$

Given an n‐length vector of training time points and biophysical parameters, the bifurcation loss function is then
(26)

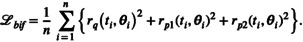




#### 3.3.2 PDE Residuals

Satisfying the PDEs represents the bulk of the networks' training. Given Equations (2) (conservation of mass) and (3) (conservation of momentum), residual functions are introduced as functions of space, time, and biophysical parameters

(27) $$r_{\text{mass}}\left(x,t,\theta \right)≔\frac{\partial \tilde{A}}{\partial t}+\frac{\partial \tilde{q}}{\partial x},$$

(28) $$r_{\mathrm{mom}}\left(x,t,\theta \right)≔\frac{\partial \tilde{q}}{\partial t}+\frac{\partial }{\partial x}\left(\frac{{\tilde{q}}^{2}}{\tilde{A}}\right)+\frac{\tilde{A}}{\rho }\frac{\partial \tilde{p}}{\partial x}+\frac{2\pi \nu \tilde{R}}{\delta }\frac{\tilde{q}}{\tilde{A}},$$

where $\tilde{q}$, $\tilde{p}$ and $\tilde{A}$ denote the predicted flow, pressure and cross‐sectional area solutions at $\left(x,t,\theta \right)$.

The loss function that captures the errors in the PDEs for an n‐length vector of training spatial locations, time points and biophysical parameters is hence
(29)

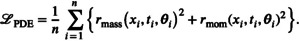




#### 3.3.3 Outflow

The loss function corresponding to the outflow boundary condition follows from the convolution relationship between flow and pressure at the end of the terminal vessels presented in Equation (12). Implementing the outflow loss requires pre‐computing a set of impedance vectors $z^{\mathrm{imp}}$ obtained using an $n^{\mathrm{imp}}$‐length training set of biophysical parameters $\theta^{\mathrm{imp}}$ generated from an LHS design which sufficiently densely covers the parameters' (patient‐specific) viable regions. The impedances are calculated at m equidistant time points in one period, $t^{\mathrm{imp}}$. For a vector of biophysical parameters θ with corresponding impedance vector z, the outflow residuals in a terminal vessel of length L are given by

(30) $$r_{\mathrm{imp}}\left(\theta ,z\right)≔\frac{1}{m}\sum_{j=1}^{m}\left\{{\left(\tilde{p}\left(L,t_{j}^{\mathrm{imp}},\theta \right)-{ℱ}^{-1}(ℱ(\tilde{q}(L,t_{j}^{\mathrm{imp}},\theta ))ℱ\left(z_{j}\right))\right)}^{2}\right\},$$

which follows from Equation (12), and where ℱ and ${ℱ}^{-1}$ denote the Fourier transform and its inverse, respectively. Then the outflow loss function for $n_{\mathrm{imp}}$‐length full batch of biophysical parameters and corresponding impedance vectors is given by

(31) $${ℒ}_{\mathrm{imp}}≔\frac{1}{n^{\mathrm{imp}}}\sum_{i=1}^{n^{\mathrm{imp}}}r_{\mathrm{imp}}\left(\theta_{i}^{\mathrm{imp}},z_{i}^{\mathrm{imp}}\right).$$

#### 3.3.4 Data‐Driven Loss

Lastly, a loss function that corresponds to a data‐driven loss term is introduced. In this case, the data represent *simulation* results of flow and pressure at the mid‐points of the vessels, obtained by numerically solving the partial differential equations for a set of biophysical parameters. For an n‐length vector of training spatial locations, time points and biophysical parameters, the data‐driven (simulation) loss is given by
(32)

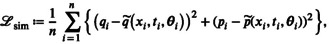

where $q_{i}$ and $p_{i}$ denote the numerical solutions of flow and pressure, respectively.

#### 3.3.5 Total Loss

Summing the individual loss terms together provides the total loss function to be minimised. Previous research has shown that weighting the individual terms appropriately can help in reaching the optima more consistently as well as more efficiently by getting there in fewer iterations [39, 40, 41, 42]:

(33) $$ℒ\left(\omega \right)≔\lambda_{\mathrm{bif}}{ℒ}_{\mathrm{bif}}+\lambda_{\mathrm{PDE}}{ℒ}_{\mathrm{PDE}}+\lambda_{\mathrm{imp}}{ℒ}_{\mathrm{imp}}+\lambda_{\mathrm{sim}}{ℒ}_{\mathrm{sim}}.$$

The goal is to find the networks' parameters which minimise Equation (33):

(34) $$\omega^{*}=\underset{\omega }{\text{argmin}}ℒ\left(\omega \right),$$

which is performed using stochastic gradient descent. More details on optimisation can be found in Section 3.8.

### 3.4 Weighting Scheme

Manually tuning the individual loss component weights in Equation (33) would prove arduous. For this reason, there has been a keen interest in investigating automatic tuning methods in the existing body of literature. The weighting scheme used in this work to set the loss terms in Equation (33) utilises the method introduced by Wang et al. [39], in which the authors investigate a potential failure mode in training PINNs caused by stiffness in gradient flow dynamics leading to unbalanced back‐propagated gradients of each loss function. An algorithm is introduced that updates the loss weights based on the ${ℒ}_{2}$ norm of the network parameters' ω gradients via a moving average using gradient statistics obtained during training every m steps

(35) $$\lambda_{i,\mathrm{new}}=\frac{\left\|\nabla_{\omega }{ℒ}_{\mathrm{bif}}\left(\omega \right)\|+\|\nabla_{\omega }{ℒ}_{\mathrm{PDE}}\left(\omega \right)\|+\|\nabla_{\omega }{ℒ}_{\mathrm{imp}}\left(\omega \right)\|+\|\nabla_{\omega }{ℒ}_{\mathrm{sim}}\left(\omega \right)\right\|}{\left\|\nabla_{\omega }{ℒ}_{i}\left(\omega \right)\right\|},$$

(36) $$\lambda_{i}={\gamma \lambda }_{i,\mathrm{old}}+\left(1-\gamma \right)\lambda_{i,\mathrm{new}},$$

for $i\in \left\{\mathrm{bif},\mathrm{PDE},\mathrm{imp},\mathrm{sim}\right\}$, and ‖⋅‖ denotes the ${ℒ}_{2}$ norm. In this work, γ was set to 0.9 and m to 5, which follows a general guideline proposed by Wang et al. [39]. The weighting scheme makes for a computationally cheap method, since the gradients are readily available as part of a single gradient descent step.

### 3.5 Multi‐Task Data‐Driven and Physics‐Informed Learning

The modelling approach investigated here is a mixed learning approach, whereby the physics‐informed loss functions presented in the previous sections are combined with a standard data‐driven approach to neural network learning. In the context of emulation, this entails conducting numerical simulations of the physical model for a set of input parameters and using the simulator's results as an additional loss objective, in addition to the physics‐informed loss terms. However, a data‐free approach may also be taken, in which case the neural networks are trained using a modified total loss (Equation 33) which does not include the data‐driven loss term (${ℒ}_{\mathrm{sim}}$). It is of interest to quantify the information gained by including the physics‐based loss terms over a data‐driven model, that is, a model trained using only ${ℒ}_{\mathrm{sim}}$ in Equation (33). The out‐of‐sample predictive accuracy of the joint data‐driven and physics‐informed emulator is compared to that of both purely data‐driven surrogate models (using deep neural networks and Gaussian Processes) and strictly physics‐informed neural networks via an ablation study in Section 4.2.2. We demonstrate that introducing even a small amount of simulator output, combined with physics‐informed constraints, yields more accurate out‐of‐sample predictions than typical data‐driven surrogate models that use several times the amount of training data. This may be especially useful in a clinical setting, for example, in the case of transfer‐learning a pre‐existing and well‐trained model to a new patient, given only a limited amount of time to run forward simulations. Further transfer learning approaches are investigated in Section 4.3.

### 3.6 Neural Network Enhancements and Activation Function

Two other methods to improve the learning of the underlying physical system by PINNs were also considered: a modified MLP that allows residual connections between the input and each hidden layer [39], and random weight factorisation (RWF) [43], a method of initialising and parameterising the weights of the hidden layers that has been shown to improve performance in some applications of PINNs [44]. A comparison of network architectures and training approaches was conducted for the 9‐vessel 5‐biophysical parameter model (Figure 2). The results are displayed in Section 4.2.2. tanh activation functions were used for all hidden layers, which is a reliable activation function in applications of physics‐informed machine‐learning [35, 44, 45].

### 3.7 Transfer‐Learning

For the analysis of real patient data in Section 4.3, the quality of emulation was evaluated through the lens of its application in a clinical setting. For all patient‐specific applications, the PINN emulator is trained solely on numerical solutions of the 1D model (using the patient‐specific geometry and inflow) together with the physics‐based loss terms. The measured flows and brachial pressures enter the workflow only through the likelihood in the Bayesian calibration step, and are not used as direct targets when optimising the PINN weights. CFD simulations were run for varying amounts of time using new patients' vascular geometries and inflow profiles, which were then used to build patient‐specific surrogate models. Instead of learning the weights and biases of the PINNs from scratch, a network trained on a previous patient was used as the starting point before fine‐tuning the weights using a stochastic gradient descent method with a small learning rate for a maximum of 30 min. In this work, the same pre‐existing PINN model was used as the base for all new patients; however, building a large set of emulators for pseudo‐patients with varying vascular geometries, boundary conditions, and physiological features and then choosing one similar to a new patient based on the features as the starting point may be an attractive route to take for larger scale applications.

### 3.8 Implementation

Optimisation of PINN network weights was performed using Adam [46], a variant of stochastic gradient descent (SGD), with a learning rate starting at $1\times 10^{-3}$ and following a cosine‐shaped decay function to a lowest value of $1\times 10^{-4}$, based on optimisation of similar problems [15, 38], and using mini‐batches consisting of 512 points.

### 3.9 Inverse Uncertainty Quantification of Biophysical Parameters

To quantify the uncertainty of the estimated biophysical parameters θ given noisy flow data, a Bayesian framework was adopted where the likelihood model used for inference was multivariate normal. The covariance structure aimed to capture the discrepancy between the fluid dynamics model and the true underlying physical model in a patient [47, 48]. This modelling choice has two benefits: firstly, it takes into account that the mathematical model used to predict flow may not be representative of the underlying system (model discrepancy), and secondly, it captures observation errors that are correlated in time (noise model mismatch). Model discrepancy may be explained by several factors and modelling choices, such as a flawed patient geometry due to errors in the MRA measurements, an incorrect inflow profile, or incorrect fixed parameter values [49]. Assuming correlated errors instead of an i.i.d. noise model was also motivated by the temporal nature of the data, that is, a measurement error affecting one time point in the period may depend on the previous measurement and affect the following. The approach taken in this study addresses both of these potential problems at once, using the GP model laid out by Kennedy and O'Hagan [48]. As indicated in Figure 2, flow data was measured in four vessels at a fixed spatial location $x_{i}$ (i=1,2,3,4) in each over $n_{T}$ time points $t_{1},\ldots ,t_{n_{T}}$ spanning one period. For each vessel, we define the vector of observed flow values as

(37) $$y_{i}={\left(y_{i}\left(t_{1}\right),y_{i}\left(t_{2}\right),\ldots ,y_{i}\left(t_{n_{T}}\right)\right)}^{T},$$

and the corresponding vector of flow values predicted by the physics‐informed neural network, parametrised by θ, as

(38) $$f_{i}\left(\theta \right)={\left(\tilde{q}\left(x_{i},t_{1},\theta \right),\tilde{q}\left(x_{i},t_{2},\theta \right),\ldots ,\tilde{q}\left(x_{i},t_{n_{T}},\theta \right)\right)}^{T}.$$

We assume for each vessel

(39) $$y_{i}\;|\;\theta \;∼\;N\left(f_{i}\left(\theta \right),\Sigma_{i}\right).$$

The correlated errors of vessel i were modelled via a zero‐mean GP prior with a Matérn 3/2 covariance function, motivated by existing works in the literature which account for model mismatch during inference using similar GPs [50],

(40) $$\Sigma_{i}=K_{\text{disc},i}+\tau_{i}I_{t_{n}},$$

(41) $${\left[K_{\text{disc},i}\right]}_{j,j^{\prime }}=\sigma_{i}^{2}\left(1+\frac{\sqrt{3}\;|t_{j}-t_{j^{\prime }}|}{\ell_{i}}\right)\exp\left(-\frac{\sqrt{3}\;|t_{j}-t_{j^{\prime }}|}{\ell_{i}}\right),$$

where $\ell_{i}$, $\sigma_{i}^{2}$ and $\tau_{i}$ are vessel‐specific length‐scale, discrepancy variance, and noise variance parameters.

Uniform proper priors over physiologically realistic parameter ranges, as shown in Tables 1 and 4, were placed on the biophysical parameters θ. For the Matérn 3/2 kernel parameters, $\text{Unif}\left(0,T\right)$ priors were placed on the lengthscale terms, where T denotes the period of a patients' circulation. Exponential priors were placed on the variance terms corresponding to each vessel, where the scale chosen was equal to the range of observed data (maximum observed—minimum observed) in that vessel. This was motivated by the idea that the mismatch between model outputs and observed data was expected to increase at a steady rate given an increase in flow magnitude. Finally, log‐normal priors were placed on the vessel‐specific observation noise terms with means of −2 and standard deviations of 1:

$$\begin{array}{ll} \;\ell_{i}∼\text{Unif}\left(0,T\right), & \sigma_{i}^{2}∼\mathrm{Exp}\left(\text{scale}=\max_{j}y_{i}\left(t_{j}\right)-\max_{j}y_{i}\left(t_{j}\right)\right),\\ \log\tau_{i}∼N\left(-2,1\right), & \theta ∼\text{Uniform}\left(\text{physiological}\;\text{bounds}\right). \end{array}$$

**TABLE 1:** 9‐vessel model parameter descriptions, and values and ranges for fixed and inferrable parameters.

Parameter	Value	Description
$f_{1}$	$2\times 10^{8}$ (fixed)	Large vessel stiffness
${\mathrm{fs}}_{1}$	$2\times 10^{8}$ (fixed)	Small vessel stiffness
$f_{2}$	[25, 45]	Large vessel stiffness
${\mathrm{fs}}_{2}$	[25, 45]	Small vessel stiffness
$f_{3}$	$\left[2\times 10^{5},9\times 10^{5}\right]$	Large vessel stiffness
${\mathrm{fs}}_{3}$	$\left[2\times 10^{5},9\times 10^{5}\right]$	Small vessel stiffness
α	[0.85, 0.94]	Structured tree scaling term
β	0.60 (fixed)	Structured tree scaling term
lrr	50 (fixed)	Small vessel length:radius ratio
$r_{\min}$	0.001 (Vessel 7), 0.0001 (rest)	Radius cut‐off of structured tree

*Note:* The stiffness parameters, which are used in Equation (5), relate either to the large vessels (f) or the small vessels which describe flow in the structured trees (fs).

The log‐likelihood for vessel i was

(42) $$\begin{array}{c} \log p\left(y_{i}|\theta ,l_{i},\sigma_{i},\tau_{i}\right)=-\frac{T}{2}\log\left(2\pi \right)-\frac{1}{2}\log|\Sigma_{i}|\\ \;-\frac{1}{2}{\left(y_{i}-f_{i}\left(\theta \right)\right)}^{T}\Sigma_{i}^{-1}\left(y_{i}-f_{i}\left(\theta \right)\right), \end{array}$$

and combining all vessels,

(43) $$p\left(\theta ,l_{i},\sigma_{i}^{2},\tau_{i}|\left\{y_{i}\right\}\right)\;\propto \;\prod_{i=1}^{M}\prod_{j=1}^{n_{\mathrm{obs}}}p\left(y_{i,j}|\theta ,l_{i},\sigma_{i}^{2},\tau \right)\;p\left(\theta \right)p\left(l_{i}\right)p\left(\sigma_{i}^{2}\right)p\left(\tau_{i}\right),$$

where the second product's subscript $j=1,\ldots ,n_{\mathrm{obs}}$ applies when there are multiple observed flow waveforms per vessel for a patient. Samples were obtained from this posterior using Hamiltonian Monte Carlo (HMC). Specifically, the No‐U‐Turn sampler [51] algorithm was used, eliminating the need to pre‐specify the number of leapfrog steps. Four chains were run in parallel with starting points sampled from the parameters' respective prior distributions. Convergence was tested by monitoring the potential scale reduction factor (PSRF) [52] until it reached 1.1, after which 2000 further samples were obtained.

## 4 Results

### 4.1 Evaluation Strategy

**FIGURE 3:**
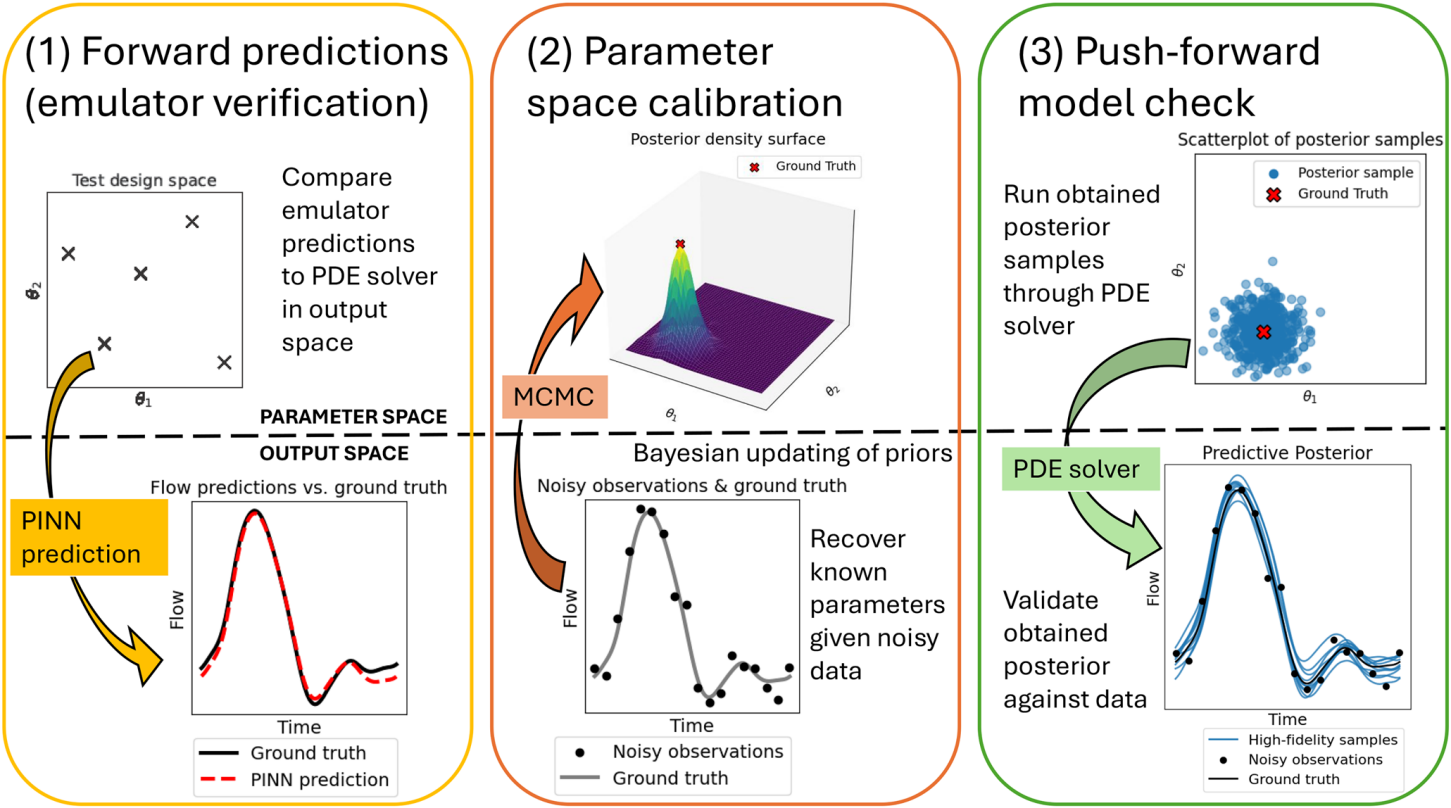
Illustration of the three levels of model evaluation. (1) Forward prediction (emulator verification): The PINN surrogate is compared to the high‐fidelity numerical solver to assess prediction accuracy in function or output space (bottom subpanel) for different combinations of physical parameters θ (note that $\theta_{1}$ and $\theta_{2}$ on the axes in the top subpanel denote two arbitrary input physical parameters). (2) Parameter‐space inference: Given noisy observational data (bottom subpanel), posterior parameter samples of the biophysical parameters are inferred (top subpanel), and their distributions are compared against known ground truth values. (3) Push‐forward validation: Posterior samples (top subpanel) are re‐inserted into the high‐fidelity solver to assess predictive accuracy in function space (bottom subpanel), comparing resulting waveforms against observed data to validate both emulator and inference performance.

The performance of the proposed PINN framework is assessed using the three‐level evaluation strategy introduced in Figure 3: (1) testing the surrogate model's accuracy in reproducing outputs from the high‐fidelity CFD solver, (2) assessing the quality of parameter inference in synthetic settings with known ground truth, and (3) evaluating predictive performance in function space via push‐forward simulations.

### 4.2 9‐Vessel Network, 5 Biophysical Parameters

The PINNs framework laid out above was first applied to a 9‐vessel network (Figure 2a) beginning in the ascending aorta. Five biophysical parameters were included as inputs to the model: stiffness parameters relating to both the 9 large vessels and the microvasculature that make up the structured tree, and an additional term that determines the shape of the structured trees (defined in Equation 13). The chosen parameter ranges are listed in Table 1. They were chosen to allow for typical wave speeds and realistic pressure ranges [53]. More details on parameter bounds, sensitivities and variable selection can be found in Paun et al. [30] and Taylor‐LaPole et al. [29].

#### 4.2.1 Training, Test and Noisy Data

$2^{14}$ combinations of spatial locations, time points, and biophysical parameter vectors were obtained using uniform sampling for space and time, and a Latin hypercube sampling (LHS) design for the biophysical parameters, with the aim being a dense coverage of all input coordinates. These vectors were then used as inputs to the loss functions, where applicable. For example, the bifurcation loss function accepts time points and biophysical parameters as inputs, whereas the PDE residuals loss function accepts spatial locations, time points and biophysical parameters. Numerical simulations of the physical model described in Section 2.2 were performed using biophysical parameters generated from the same LHS design consisting of roughly 5000 points. The outputs (flow and pressure) of the numerical solver consisted of 512 time points at the midpoint of each vessel. These input–output pairs were then subsampled using the following training data set sizes: {128, 512, 1024, 2048, 4096}, to be used in the data‐driven loss (Equation 32). Each set of simulation data was then used to build separate surrogate models.

**FIGURE 4:**
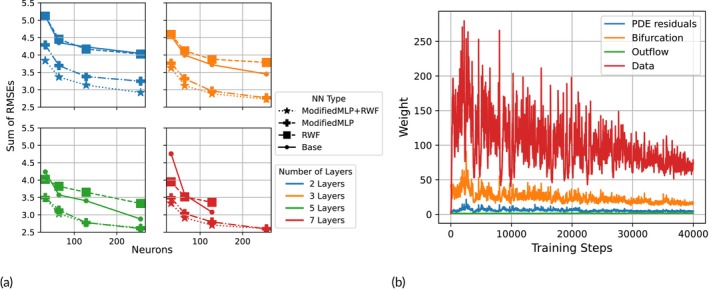
(a) Comparison of the models' predictive accuracy on an out‐of‐sample test set: Number of neurons per hidden layer versus sum over 9 vessels of mean RMSE scores. The colours and shapes refer to the number of hidden layers and neural network properties, respectively. Both the number of neurons per hidden layer and the number of hidden layers have a strong impact on predictive performance, with performance plateauing when using at least 5 hidden layers, 256 neurons per layer, and the modified MLP architecture. Note that values for the 7‐layer 256‐neuron Base and RWF models were left out, as they were not able to converge during training, and further investigation of appropriate learning rates was considered outside the scope of this work. (b) Traceplots describing the evolution of loss component weights over the course of training the best performing model in the architecture comparison.

A test set with 128 input–output numerical simulation pairs was created by randomly sampling and removing from the training set, as outlined in the previous paragraph. Both training and test sets used only 20 time‐wise equidistant flow data points over one period from the midpoints of each vessel instead of the full 512, to mirror what the data‐driven surrogates would typically use (see Section 4.2.2). Predictive accuracies of the trained PINN models are compared to purely data‐driven methods in Section 4.2.2. For each vessel and each test simulation, the vessel‐specific test loss was defined as the root mean squared error (RMSE) of predictions against the test data:

(44) $${\text{RMSE}}_{i}=\sqrt{\frac{1}{20}\sum_{j=1}^{20}{\left(q_{\mathrm{ij}}-{\tilde{q}}_{\mathrm{ij}}\right)}^{2}},$$

where the indices i,j denote the ith element of the test set and the jth time point, respectively. The total loss was calculated by summing the RMSE scores of all individual vessels, and the distributions of ${\text{RMSE}}_{i}$ over the 128 test simulations are shown in Figure 4a.

To give a more concrete sense of emulator accuracy in physical units and relative terms, mean absolute error (MAE), maximum absolute error, and a relative $L^{2}$ error for flow and pressure were computed for each vessel and test simulation. These are defined for flow as

(45) $${\mathrm{MAE}}_{i}=\frac{1}{20}\sum_{j=1}^{20}|q_{\mathrm{ij}}-{\tilde{q}}_{\mathrm{ij}}|,\;{\text{MaxAE}}_{i}=\max_{1\leq j\leq 20}|q_{\mathrm{ij}}-{\tilde{q}}_{\mathrm{ij}}|,$$

and

(46) $$\mathrm{rel}\;{ℒ}_{i}^{2}=\frac{\sqrt{\left(\sum_{j=1}^{20}{\left(q_{\mathrm{ij}}-{\tilde{q}}_{\mathrm{ij}}\right)}^{2}\right)}}{\sqrt{\left(\sum_{j=1}^{20}q_{\mathrm{ij}}^{2}\right)}}.$$

The same quantities are defined for pressure by replacing $q_{\mathrm{ij}}$ and ${\tilde{q}}_{\mathrm{ij}}$ with $p_{\mathrm{ij}}$ and ${\tilde{p}}_{\mathrm{ij}}$.

In order to test the PINNs' ability to perform uncertainty quantification, 4 sets of synthetic, noisy flow data were generated using biophysical parameters sampled from their viable ranges, as presented in Section 4.2. The ground truth data consisted of 20 time‐wise equidistant flow measurements in one period, obtained using the CFD solver introduced in Section 2.2 at the midpoints of Vessels 3, 5, 6 and 7. Correlated errors were produced and added to the CFD solutions by sampling from the zero‐mean GP prior outlined in Section 3.9. The variance parameter $\sigma_{i}^{2}$, i∈3,5,6,7, was set to be vessel‐specific to aim for similar signal‐to‐noise ratios, while the same lengthscale parameter l and noise variance τ was shared between all vessels for generating the data. These values were considered known and fixed during inference in order to isolate the performance of the emulator in recovering the physical parameters. While this would not be realistic in a clinical setting, it was decided upon for this synthetic validation study to ensure a controlled and interpretable comparison to ground‐truth posteriors.

#### 4.2.2 Emulation Accuracy

**FIGURE 5:**
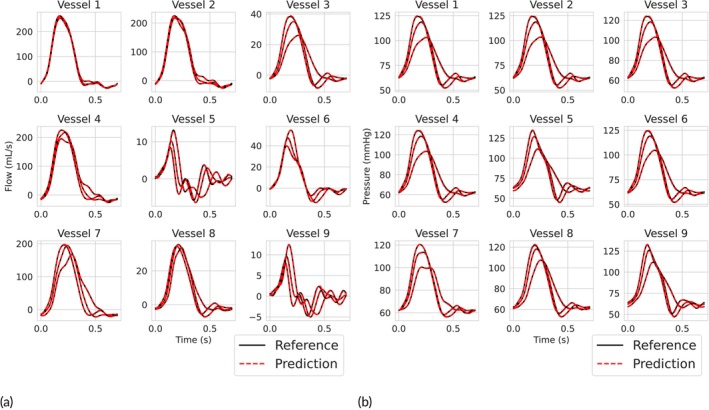
Flow and pressure waveform predictions versus ground truths for three different vectors of physical parameters in the test set. The predicted flow (a) and pressure (b) were obtained using the best performing model in the architecture comparison.

A comparison of architectures was conducted using a simulation batch consisting of 128 input and output pairs. The performance with respect to out‐of‐sample predictive accuracy of the various architectures is presented in Figure 4a. This figure illustrates the importance of deeper networks, with performance further improving as more neurons are added. Figure 5 displays predicted pressure and flow waveforms at the midpoint of each of the 9 vessels of the 5‐layer, 128‐neuron PINN model utilising both the modified MLP and RWF, and the corresponding ground truth obtained via CFD simulations for 3 vectors of biophysical parameters included in the test set.

**FIGURE 6:**
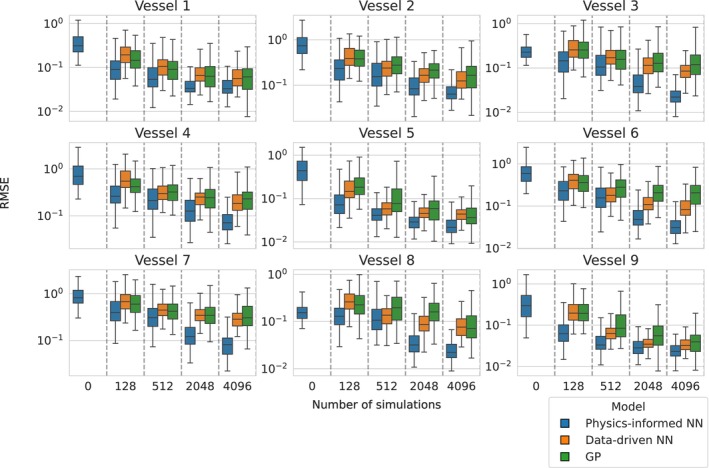
Comparison of physics‐informed neural networks, data‐driven neural networks and Gaussian processes' predictive performances in the 9‐vessel model given by RMSE error scores (displayed on the log scale) on an out‐of‐sample test set of 128 simulations using varying amounts of data to train each model (displayed on the *x*‐axis). The extra information provided by the physics‐informed constraints contained within the PINNs resulted in lower averages (59% and 49% improvement in mean RMSE across all vessels and number of simulations over GPs and DDNNs, respectively) across the entire test set compared to either of the other two methods when trained on the same amount of simulation data. Training the PINNs using 512 forward simulations yields comparable performance to the data‐driven methods trained using 8 times as many (4096) simulations.

To compare the emulation performance of PINNs versus standard data‐driven surrogate modelling techniques, deep neural networks and Gaussian processes were assigned to each of the 9 vessels, which were trained on varying amounts of simulation data. This corresponds to the first level of evaluation in Figure 3, focusing on forward prediction accuracy of the surrogate model. The data‐driven methods were trained using vectors consisting of 20 equidistant points of the original 512 stretching across the whole temporal domain. This was chosen based on a typical amount of patient data available in a clinic setting for inference (see Section 4.3) [29]. The data‐driven neural networks (DDNNs) were trained in a multi‐output fashion with 20 output neurons, corresponding to each time point, whereas 20 independent Gaussian processes were fit using the numerical simulation flow output at each of the time points. The total data budget was fixed for all models. Neural networks used a 90:10 train/validation split of the simulation data for early stopping and underwent a grid search to find the optimal architecture, while GPs were trained using the full set. This design ensured that no method benefitted from access to more simulation outputs. Accompanying PINN models were fit using the same amount of numerical simulation output as the data‐driven methods. The architecture chosen was based on the comparison above: 5 hidden layers with 128 neurons per layer, and utilising both the modified MLP and RWF, which provided a good balance between predictive performance and training time.

The same test set was used as in the architecture analysis. RMSE scores for each of the 20‐length test set vectors were obtained using the fully trained DDNNs, PINNs and GPs, and the results are summarised in Figure 6. The PINNs averaged better scores than the data‐driven methods across all tests (48% ± 16% and 40% ± 15% improvement in mean RMSE (± one standard deviation) across all vessels and number of simulations over GPs and DDNNs, respectively) due to having more information of the underlying physical process naturally built into them. Using as few as 512 simulations provided roughly the same level of predictive performance as the competing data‐driven methods achieved when using 4096 simulations. This stark difference describes the impact that the physics‐based loss function has on out‐of‐sample predictive accuracy. Table 2 provides further metrics for the PINN trained with 128 simulations, displaying the mean and standard deviation of the MAE and relative $L^{2}$ error across the 128 test simulations, together with the corresponding maximum absolute error. Maximum absolute errors for pressure are within a unit across the network, showing strong performance.

**TABLE 2:** Flow and pressure mean (standard deviation) error scores (mean and max absolute, and relative L2) across the test set per vessel for the PINN model trained using 128 simulations.

Vessel	Flow MAE	Flow Max AE	Flow Rel L2	Pressure MAE	Pressure Max AE	Pressure Rel L2
1	0.089 (0.066)	0.232 (0.172)	0.001 (0.001)	0.185 (0.133)	0.545 (0.398)	0.003 (0.002)
2	0.222 (0.163)	0.641 (0.470)	0.003 (0.002)	0.182 (0.132)	0.537 (0.404)	0.003 (0.002)
3	0.126 (0.084)	0.365 (0.258)	0.010 (0.007)	0.181 (0.129)	0.530 (0.384)	0.003 (0.002)
4	0.279 (0.201)	0.805 (0.614)	0.004 (0.003)	0.176 (0.130)	0.511 (0.394)	0.003 (0.002)
5	0.089 (0.102)	0.248 (0.291)	0.030 (0.032)	0.242 (0.184)	0.700 (0.567)	0.004 (0.003)
6	0.220 (0.152)	0.675 (0.442)	0.016 (0.010)	0.182 (0.135)	0.526 (0.405)	0.003 (0.002)
7	0.365 (0.230)	1.042 (0.691)	0.006 (0.004)	0.136 (0.098)	0.392 (0.295)	0.002 (0.002)
8	0.110 (0.063)	0.332 (0.200)	0.010 (0.006)	0.177 (0.130)	0.499 (0.371)	0.003 (0.002)
9	0.081 (0.089)	0.200 (0.179)	0.030 (0.033)	0.253 (0.193)	0.705 (0.537)	0.004 (0.003)

*Note:* Mean and max absolute error scores are in the corresponding units (mL/s and mmHg), while relative L2 is in percentage points.

#### 4.2.3 Inverse Uncertainty Quantification of Biophysical Parameters Using the Emulator

**FIGURE 7:**
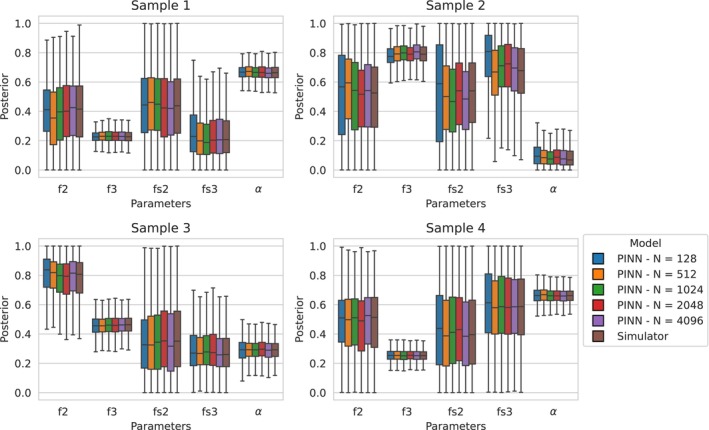
Boxplots comparing the PINNs' and ground truth marginal posterior quartiles. Note that the outputs have been scaled to lie between 0 and 1 for ease of comparison. The models' boxplots are in the following order: *N =* 128, 512, 1024, 2048, 4096, and finally the ground truth obtained via the numerical solver. There is not much of a difference in posterior quartiles when using more than or equal to 512 runs of the numerical solver to train the models.

Given the noisy flow observational data generated as outlined in Section 4.2.1, ground truth posteriors were obtained using Bayesian inference, where forward runs of the numerical CFD solver were used within a Markov Chain Monte Carlo (MCMC) sampling procedure to evaluate the likelihood at each parameter setting. This approach provides a high‐fidelity posterior against which the PINN‐based posteriors can be compared. This analysis addresses the second level of model assessment shown in Figure 3, examining how well the inferred parameter posterior distributions align with the ground‐truth posterior. Due to the computational cost of repeatedly solving the full 1D fluid dynamics model, this procedure required a timescale on the order of days. In contrast, the PINN emulators produced comparable posteriors in under an hour, highlighting the practical advantage of emulation in time‐constrained settings. Boxplots of the marginal posteriors are displayed in Figure 7, and KL‐divergence scores summarising the differences between the PINN and ground truth posteriors are given in Table 3.

**TABLE 3:** Summary of KL‐divergences between the ground truth posterior obtained via simulations and the posteriors found using PINNs with varying amounts of simulation data used during training.

	128	512	1024	2048	4096
Sample 1	0.072	0.033	0.022	0.012	**0.0093**
Sample 2	0.46	0.064	0.057	0.051	**0.032**
Sample 3	0.062	0.069	0.12	0.043	**0.033**
Sample 4	0.059	0.015	0.0101	0.016	**0.009**

*Note:* The KL‐divergences tend to decrease in size when using PINNs trained on more simulation data. The posteriors which resulted in the smallest KL‐divergence are in bold.

### 4.3 17‐Vessel Network, 10 Biophysical Parameters

For the 17‐vessel network, the arteries are divided into 4 groups by location, with each group having a unique vessel stiffness parameter, thereby increasing the number of parameters in the model from 5 to 10 (Equation 14, see Table 4 for inferrable parameter bounds and descriptions). This allows for more flexible modelling of blood circulation, with the challenge of a larger parameter space required to be accurately emulated. The modelling methods closely follow the study conducted by Taylor‐LaPole et al. [29], including variable selection and most of the parameter bounds.

**TABLE 4:** Patient 1: 17‐vessel model parameter descriptions, values and ranges for fixed and inferrable parameters.

Parameter	Value	Description
$f_{1}$	$2\times 10^{7}$	Large vessel stiffness (fixed)
${\mathrm{fs}}_{1}$	$2\times 10^{7}$	Small vessel stiffness (fixed)
$f_{2}$	35	Large vessel stiffness (fixed)
${\mathrm{fs}}_{2}$	$\left[16,88\right]$	Small vessel stiffness
$f_{\left(3,\cdot \right)}$	$\left[4\times 10^{5},2.2\times 10^{6}\right]$	Large vessel stiffness
${\mathrm{fs}}_{\left(3,\cdot \right)}$	$\left[3.52\times 10^{4},1.936\times 10^{5}\right]$	Small vessel stiffness
α	$\left[0.90,0.94\right]$	Structured tree scaling term
β	0.60 (fixed)	Structured tree scaling term
lrr	50 (fixed)	Small vessel length:radius ratio
$r_{\min}$	0.001 (Vessel 7), 0.0001 (rest)	Radius cut‐off of structured tree

*Note:* There are four unique, group‐specific parameters for $f_{3}$ and ${\mathrm{fs}}_{3}$, and the empty subscript replaced with a dot refers to the parameter bounds being shared between groups.

In this study, bounds for these parameters were estimated using Equation (5), inserting the patient's pulse pressure obtained from systolic and diastolic cuff pressure measurements and typical expansion rates of arterial cross‐sectional area reported in the literature. For example, Thijssen et al. [54] and Green et al. [55] noted that the typical arterial expansion ranges from 3% to 10% depending on the artery.

#### 4.3.1 Uncertainty Quantification of Vessel‐Stiffness Parameters

For the 17‐vessel model, the parameters to be inferred using the likelihood model defined in Section 3.9 were: 10 biophysical parameters (4 large and 5 small vessel stiffness parameters and a structured tree scaling parameter), 8 parameters (2 per vessel) defining the vessel‐specific Matérn 3/2 kernel used for the covariance matrices to model correlated error/model discrepancy structure, and 4 vessel‐specific observation noise parameters. Results for Patient 1 are discussed in the following section, and results for the 3 further patients can be found in Supporting Information.

#### 4.3.2 Patient 1

**FIGURE 8:**
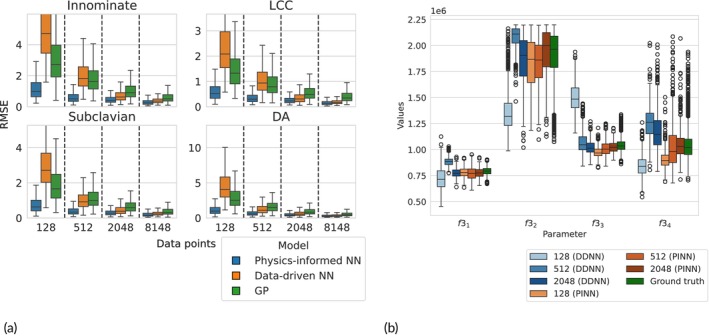
Comparing predictive and inference performance of physics‐informed and data‐driven neural networks trained on varying amounts of simulator runs: (a) RMSE scores on a held‐out test set of flow data from vessel midpoints generated using 512 different vectors of biophysical parameters. Similarly to the 9‐vessel network, the PINNs in the 17‐vessel network exhibit better predictive performance compared to the other methods when trained on the same amount of data. This is explained by the fact that PINNs have more information about the underlying process built into them. Notably, the PINNs require roughly 4 times fewer simulations to achieve similar predictive accuracy as the data‐driven methods trained on 2048 simulations. (b) Marginal posterior quartiles of the large vessel stiffness parameters inferred using DDNNs and PINNs as surrogate models trained with varying amounts of simulation training data. The posteriors obtained using the PINNs and the DDNN with the most training data appear to approximate the ground truth posterior quite accurately, apart from bias in the posterior mean of $f3_{4}$. In contrast, the DDNNs with the least training data cannot approximate the posterior. The PINNs' outputs are regularised strongly given input stiffness parameters due to the relationship between area and pressure in Equation (5).

Physics‐informed neural networks, data‐driven neural networks, and Gaussian processes were implemented and trained using varying amounts of simulation data for different vectors of physical parameters. Simulations were run in parallel and performed using Dual Intel Xeon Gold 6138 processors. Table 5 displays the time (in minutes) to run 4 different batches of input data. Figure 8a highlights the out‐of‐sample predictive performance of each of the emulation methods trained on each of the batches of simulation data.

**TABLE 5:** Time taken to run varying amounts of simulations.

Number of simulations	128	512	2048	8148
Time taken in minutes	30	120	480	1920

**FIGURE 9:**
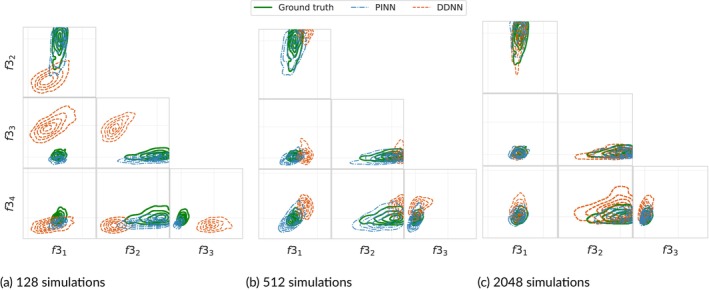
Pairplots and densities comparing posteriors obtained via PINNs and DDNNs using varying amounts of training data (128, 512 and 2048). The green densities and contours correspond to the ground truth posterior and hence remain constant between plots.

As outlined in the evaluation strategy in Figure 3, we assess the inference performance of the emulators not only in parameter space but also in output space via push‐forward validation. Figure 8b shows the marginal posterior distributions inferred using both data‐driven emulators and physics‐informed neural networks (PINNs), each trained on simulation datasets of increasing size. To assess how well each model captured the true posterior distribution under noisy observational data, we computed a ground‐truth posterior using the numerical solver embedded within an adaptive Metropolis algorithm. Achieving sufficient convergence across all parameters (PSRF < 1.1) required slightly over a week of computation. As shown in Table 6, PINNs achieved lower KL‐divergences to the ground‐truth posterior than their data‐driven counterparts, despite requiring roughly four times fewer training simulations to reach comparable predictive accuracy on the test set. Contour plots of posterior samples of stiffness parameters corresponding to each of the four vessel group obtained using the PINNs and DDNNs with the varying training set sizes and the numerical solver are displayed in Figure 9.

**TABLE 6:** KL‐divergences between the ground truth posterior obtained using the numerical solver and the posteriors found using both DDNNs and PINNs, with varying amounts of simulation data used during training.

Number of simulations	128	512	2048
DDNN	18.46	5.94	1.71
PINN	2.11	1.44	1.13

**FIGURE 10:**
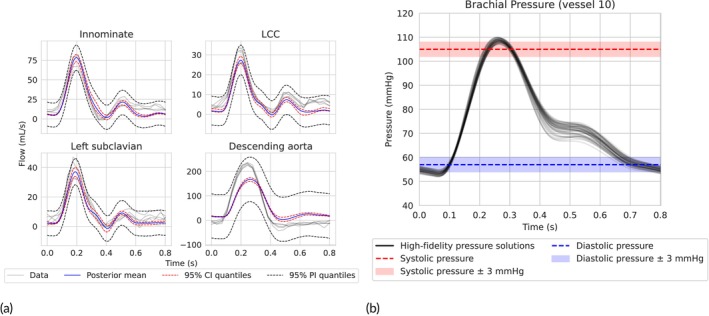
Patient 1 inference results: (a) 95% posterior predictive credible and prediction intervals in each of the four vessels used for inference. (b) 95% posterior predictive credible interval in the brachial artery. Pressure predictions based on the inferred biophysical parameters show relatively good agreement with the measured systolic and diastolic pressures. The shaded regions indicate ±3 mmHg measurement error.

Figure 10 displays posterior predictive intervals for both flow and pressure. The posterior samples were subsampled down to 50 sets of physical parameters, which were then inserted into the PDE solver to obtain the set of high‐fidelity pressure solutions for the inferred posterior displayed in Figure 10b. This push‐forward validation corresponds to the third level of evaluation in Figure 3, where we assess whether posterior uncertainty over parameters translates into plausible uncertainty in predicted pressure waveforms. The flow credible intervals were generated by passing each of the samples from the inferred posterior distribution through the trained PINN model. This push‐forward step propagates parametric uncertainty into the output space, producing distributions over predicted physiological quantities such as flow and pressure waveforms.

In Vessels 3, 5 and 6, the predicted flow envelopes show close agreement with the observed 4D‐MRI data. In contrast, Vessel 7 exhibits a discrepancy between predicted and observed waveforms, which may be attributed to model mismatch, measurement noise, or dynamics missing from the model which are specific to that location. In the absence of invasive pressure measurements, we use cuff‐based brachial pressure as a surrogate validation target. While this is an indirect metric, it reflects a clinically meaningful endpoint and provides a practical means of validating output‐space predictions. The predicted pressure distribution at the midpoint of the brachial artery (Vessel 10; see Figure 2b) demonstrates the model's ability to estimate central blood pressure non‐invasively. Dashed red and blue lines represent the measured systolic and diastolic pressures, respectively, with the shaded band indicating a ±3 mmHg tolerance corresponding to the reported accuracy of clinical cuff‐based measurements [56, 57]. The predicted range lies well within this band, supporting the model's potential for practical use in pressure estimation.

All predictive simulations used posterior samples obtained from a PINN trained on 512 simulation points. The full inference workflow required approximately three hours per patient: two hours to generate simulation data (see Table 5), 30 min for patient‐specific fine‐tuning via transfer learning, and 30 min for posterior inference.

#### 4.3.3 Patients 2–4

To test the generalisability of the method across a broader cohort, we applied the same workflow to three additional patients. In all three cases, the predicted brachial pressure intervals aligned well with the measured systolic and diastolic values, falling within the ±3 mmHg clinical tolerance. These results, shown in the Supporting Information, provide further evidence that the proposed approach can generalise across patients and produce clinically relevant predictions with minimal tuning. Additionally, compliance distributions in the four vessels where flow data was measured are reported in Supporting Information. The values match the range of inferred compliances in the original study.

#### 4.3.4 Information Provided by Physics‐Based Constraints

Based on the results of the previous sections, the constraints provided by the physics‐based loss functions have a significant impact on creating an accurate predictive model. In this section, an attempt is made to quantify the amount of information that is provided by the physics‐informed loss functions in finding the solutions to the physical problem. The predictive performance in Section 4.2.2 (specifically Figure 6) highlighted that introducing even a small amount of data in the form of solutions to the physical problem for a small set of physical parameters improved predictive performance by a significant amount, despite the remaining loss functions being the same. In other words, introducing the solutions obtained via the numerical solver as a loss function may aid the neural networks in minimising the remaining loss functions, and hence finding the solutions to the PDEs.

**FIGURE 11:**
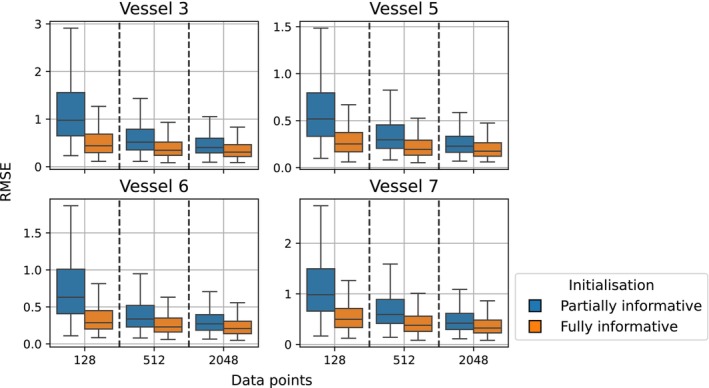
Comparing RMSE scores on an out‐of‐sample test set given different PINN initialisations. The boxplots in orange display scores obtained by models which used the final weights of the model trained on 8148 data points as their starting points, whereas the boxplots in blue display scores of models initialised on a separate patient's (less informative) model weights.

To validate this idea, a second round of PINNs were fit to the 17‐vessel network corresponding to Patient 1 (Section 4.3.2). Instead of using an uninformative set of initial weights as the starting point for fitting the model, the weights were initialised at the final weights of a well‐trained model, that is, the emulator which was trained using the largest amount of numerical solver data (see Figure 8a, 8148 runs of the numerical solver using different vectors of physical parameters each time). The predictive performance of the trained models after using the best available initialisations are compared to the previous results (presented in Section 4.3.2) in Figure 11. The models trained on fewer data retain much of the information provided by the initialisation, as the physical constraints provided by the loss functions are still being met. In summary, the physics‐informed loss functions offer a wealth of information for obtaining solutions to the PDEs. One of the drawbacks of the PINNs compared to the data‐driven models, however, is an increase in training time due to the much more complex loss surface resulting from the sum of individual losses. The weighting scheme introduced in Section 3.4 was one attempt to balance the loss terms and allow for efficient training. For the 1D fluid dynamics problem of branching networks tackled in this work, the focus moving forward may lie on finding an ideal optimisation routine to obtain the encouraging results in Figure 11 without relying on a fully informative initialisation.

## 5 Discussion

### 5.1 Physics‐Informed Emulator

In this work, a method for implementing physics‐informed neural networks as emulators of a complex fluid dynamics problem involving a system of vessels around the heart was introduced and compared to standard emulation techniques. In comparative experiments the physics‐informed surrogate consistently outperformed standard data‐driven emulators in both forward prediction accuracy and inverse UQ while using substantially fewer high‐fidelity simulations. The PINN emulator achieved comparable RMSE performance when trained on 512 CFD simulations whereas the purely data‐driven deep neural networks and GP surrogates required 4096 simulations to reach a similar error level (8× larger training budget). Across the tested vessels the PINN models yielded mean RMSE improvements on the order of 40%–48% relative to DDNNs and GPs, and produced posteriors with low KL divergence to simulator‐based ground truth (see Table 3 and Figures 7 and 8b). These quantitative gains highlight that embedding conservation laws and boundary constraints directly into the learning objective substantially improves out‐of‐sample generalisation. Compared to other applications in the field which use physics‐informed models to infer physiological parameters from simplified or smaller‐scale models, we extend the framework to a larger vessel network with multiple interconnected arteries, vessel compliance, and structured outflow boundary conditions. For instance, Naghavi et al. [58] employ a closed‐loop 0D circulation model of the left ventricle and surrounding components aorta, peripheral arteries, vena cava, and left atrium, using PINNs to estimate contractility parameters from single‐beat pressure–volume waveforms. While accurate and fast, their approach does not capture spatial heterogeneity or flow in multiple vessels. Similarly, Jara et al. [59] use a 1D Navier–Stokes model together with MRI imaging to estimate pulmonary artery pressures, which brings in spatial flow/area data but remains limited in network complexity and number of vessels (three vessels with one bifurcation). In contrast, we model a 17‐vessel network with full enforcement of mass conservation and flow continuity, and crucially, infer both systolic and diastolic pressures in unobserved vessels (i.e., vessels lacking direct pressure measurements).

A practical concern is whether a PINN‐based approach remains tractable as the vascular network grows. The architecture used throughout the paper, 5 hidden layers with 128 neurons per layer with the modified MLP, has a total of 68,096 parameters. In our formulation, a separate fully connected network is assigned to each large vessel, all sharing the same architecture. Consequently, both the total number of trainable parameters and the associated training cost scale linearly with the number of vessels. For example, moving from 9 to 17 vessels increased the total parameter count from $6.13\times 10^{5}$ to $1.16\times 10^{6}$, with a proportional change in training time and no degradation in optimisation stability due to the loss scaling scheme which has trivial complexity (Section 3.4). Cross‐vessel coupling enters only through bifurcation constraints, whose number is also linear in network size. Importantly, once trained, inference for biophysical parameters remains constant‐time (depending only on the number of vessels data in which data was observed), as predictions are vessel‐specific and do not depend on the total network size. This linear scaling suggests that extending the framework to larger arterial networks is computationally feasible, especially given the natural parallelism of the branching structure and the potential for transfer learning demonstrated in this study.

Several alternative strategies have been proposed concerning patient‐specific parameter inference in haemodynamic networks. A common approach is surrogate‐assisted Bayesian sampling, for example GP emulators used to approximate a likelihood in MCMC to accelerate posterior exploration [10, 50], or as emulators of the forward model outputs themselves [60, 61, 62]. For example, Schiavazzi et al. [60] use a GP surrogate for virtual Fontan surgery to propagate uncertainty in geometric and boundary‐condition parameters through 3D haemodynamic simulations for single‐ventricle palliation. Their surrogate, however, is constructed in a very low‐dimensional parameter space and targets a small set of scalar outputs, whereas the present work proposes an emulator for time‐resolved 1D haemodynamics over a higher‐dimensional biophysical parameter space. More recently, graph neural networks have been proposed as surrogates for cardiovascular simulations, such as left ventricular displacement by Dalton et al. [63, 64] and arterial blood flow by Pegolotti et al. [65]. While the GNN‐based models achieve very low errors for fixed anatomies and boundary conditions, extending them to emulate solutions over a biophysical parameter space (e.g., vessel wall stiffness and structured‐tree outflow parameters) would require explicitly embedding these parameters into the graph representation. Our PINN formulation instead accepts them as inputs at each prediction, while enforcing the governing 1D PDEs and network constraints. In parallel, amortised simulation‐based inference methods have been developed for lumped‐parameter haemodynamic models. For example, the InVAErt framework [66, 67] trains a conditional generative model intended to approximate θ∣yand enables fast approximate posterior sampling and identifiability analyses across many patients. However, that work does not explicitly assess the accuracy of the inferred posterior in parameter space. These approaches also require specifying a noise/likelihood model a priori in order to generate synthetic training pairs. In the present work we deliberately retain an explicit Bayesian calibration step with a flexible, GP model discrepancy term, allowing the observation noise and model mismatch to be inferred jointly with θ rather than fixed at training time. Ensemble‐based methods such as the ensemble Kalman filter/inversion [68] require forward evaluations of the full model for every ensemble member at each assimilation step; this is computationally inexpensive for 0D or lumped‐parameter haemodynamic models and thus commonly used in clinical pipelines, but it becomes prohibitive for expensive 1D/3D solvers unless paired with a fast surrogate. Conversely, adjoint‐based gradient approaches [69, 70] are attractive because they yield gradients at a cost roughly independent of parameter dimension, but deriving and implementing either the continuous or discrete adjoint for complex coupled blood flow models is non‐trivial and often a significant engineering effort. PINNs sit between these classes: by embedding the PDE structure they retain computational efficiency (fewer numerical solver runs required to achieve a similar level of accuracy to a data‐driven surrogate) while enabling full posterior inference using the full‐order model (FOM). In this study the PINN‐based emulator reduced the simulator budget needed for reasonable posterior recovery by roughly an order of magnitude compared with purely data‐driven surrogates, making it an attractive compromise for networks where both fidelity and computational cost matter.

### 5.2 Model Parameters and Predictions

Parameter inference was performed using flow data from four patients, and the emulated fluid dynamics model's pressure predictions were shown to accurately match systolic and diastolic cuff pressure measurements as shown in Figure 10b and in Supporting Information. Inference was performed first by transfer‐learning an existing model to the new patient, reducing the time needed to fit the model and allowing for rapid parameter estimation. The Supporting Information contains further comparisons to the point estimates of the stiffness values for the DORV patients obtained in a previous study by Taylor‐LaPole et al. [29]. The posterior flow predictions displayed in Figure 10a as well as in Supporting Information indicate a strong ability to match most reflective waves as well as peak flows. However, even with patient‐specific geometry and inflow, we do not assume that the 1D model can reproduce all measured 4D‐MRI flows exactly. The GP discrepancy term in the likelihood is intended to capture structural model error and noise‐model mismatch arising from, for example, uncertainty in segmented radii and lengths [49], imperfect registration between MRI planes and 1D vessel locations [71], simplified outflow boundary conditions, and the reduction from 3D haemodynamics to a 1D elastic‐tube model.

To provide a physiologically meaningful comparison we also translate inferred f‐parameters into effective compliance as in Section 2.2.3 ($C=\frac{3A_{0}r_{0}}{2\mathrm{Eh}}$). The results for all four patients are shown in the Supporting Information.

### 5.3 Limitations

The inference framework presented in this work has several limiting factors. The PINN model requires patient‐specific inputs, including vascular geometry and inflow waveforms, which are themselves obtained from data subject to measurement error. In this study, these quantities were treated as known and fixed, meaning that uncertainty in their measurement was not propagated through the inference procedure. While this may not strongly bias the inferred parameters in the absence of systematic error, it can lead to an underestimation of posterior uncertainty. Including the inflow function or geometry parameters as inputs into the model, using, for example, neural operators [72], would allow their uncertainty to be taken into account.

Furthermore, the parameter ranges that were used in building the emulator for each of the four patients used expert knowledge from a previous study by Taylor‐LaPole et al. [29]. Extending the emulator to a new patient may require additional expert knowledge, or a new method to ascertain nominal parameter values and ranges. Lastly, the framework laid out in this paper was only applied to four DORV patients and would benefit from a larger case–control study including both healthy (control) and diverse pathological cohorts to assess the robustness of the inference pipeline in realistic clinical settings.

## 6 Conclusion

Physics‐informed machine learning is a powerful tool for incorporating valuable information about an underlying process into a model. When clinicians are making surgical decisions when constructing the Fontan circuit, the limited time available to create a patient‐specific model must be used efficiently. The PINNs presented in this paper were shown to outperform two widely used, purely data‐driven surrogate modelling methods, deep neural networks and Gaussian Processes, in terms of predictive accuracy (output space) and inverse UQ (parameter space) in two different models of systemic circulation, requiring up to eight times fewer CFD simulations to reach the same level of predictive accuracy. A potential avenue for future work is to use the PINNs as efficient emulators to quickly infer pressure waveforms in the aorta and use these as initial conditions in complex 3D Navier–Stokes simulations.

## Author Contributions

**William Ryan:** conceptualisation, methodology, formal analysis, software, visualisation, investigation, writing – original draft, writing – review and editing. **Alyssa Taylor‐LaPole:** conceptualisation, visualisation, software, writing – review and editing. **Mette Olufsen:** conceptualisation, visualisation, software, formal analysis, writing – review and editing. **Dirk Husmeier:** conceptualisation, methodology, formal analysis, writing – review and editing. **Vladislav Vyshemirskiy:** conceptualisation, methodology, formal analysis, writing – review and editing.

## Funding

This work was supported by Engineering and Physical Sciences Research Council (EPSRC), grant reference no. EP/T017899/1; National Science Foundation (NSF), grant reference no. DGE‐2137100, DMS‐2231482, and 2342344; Additional Ventures, award number 1449780.

## Conflicts of Interest

The authors declare no conflicts of interest.

## Supporting information


**Data S1:** cnm70147‐sup‐0001‐Supplementary.pdf.

## Data Availability

Waveform and geometry data for DORV patients included in this study can be found at https://doi.org/10.5061/dryad.zpc866tj0 and in Supporting Information of Taylor‐LaPole et al. [29].
