# Supplementary material for: Physics‐Informed Emulation of Systemic Circulation for Fast Parameter Estimation and Uncertainty Quantification
Source: Int J Numer Method Biomed Eng. 2026 Feb 13;42(2):e70147. doi: 10.1002/cnm.70147 (PMC12905477; doi:10.1002/cnm.70147)
Supplement: Supplementary file 1 — Data S1: cnm70147‐sup‐0001‐Supplementary.pdf. [file CNM-42-e70147-s001.pdf]

Supplementary Material for: Physics-informed emulation of systemic circulation for fast parameter estimation and uncertainty quantification

William Ryan, Alyssa Taylor-LaPole, Vladislav Vyshemirsky, Mette S. Olufsen, Dirk Husmeier

A DORV patient 1 - comparison to original study

Figure 1a displays pairplots of the posterior distributions of the 4 large vessel parameters inferred for Patient 1, obtained using both the physics-informed neural networks introduced in the main text and the numerical solver for forward predictions during MCMC. Additionally, the point estimates found in the original study by Taylor-LaPole et al. [1] are overlaid with a red cross.

B DORV patients 2, 3 and 4 - Inference results

Table 1 displays the means (standard deviations) of the inferred posterior distributions for the 10 inferrable parameters for each of the 4 patients, while the posterior flow and pressure predictions are displayed in Figures 2, 3 and 4.

Table 1: Posterior parameter means (standard deviations) for each of the 4 patients.

Parameter	Patient 1	Patient 2	Patient 3	Patient 4
$f_{3,1}$	7.71e+05 (6.03e+04)	5.58e+05 (5.85e+04)	9.18e+05 (1.73e+05)	5.29e+05 (5.15e+04)
$f_{3,2}$	1.84e+06 (2.20e+05)	1.10e+06 (2.29e+05)	1.15e+06 (1.95e+05)	1.31e+06 (1.73e+05)
$f_{3,3}$	1.01e+06 (7.19e+04)	2.13e+06 (7.50e+04)	2.49e+06 (2.11e+05)	1.41e+06 (4.13e+05)
$f_{3,4}$	1.04e+06 (2.34e+05)	2.15e+06 (4.22e+04)	2.63e+06 (1.17e+05)	1.46e+06 (1.92e+05)
f_{s2}	17.6 (0.969)	17.4 (1.21)	17.4 (1.28)	71.2 (12.6)
$f_{s3,1}$	8.37e+04 (1.01e+04)	2.08e+05 (7.05e+03)	2.45e+05 (2.88e+04)	2.48e+05 (7.48e+03)
$f_{s3,2}$	7.91e+04 (3.03e+04)	8.27e+05 (2.04e+05)	1.03e+06 (9.07e+04)	1.25e+06 (6.41e+04)
$f_{s3,3}$	1.55e+05 (3.56e+04)	1.03e+06 (6.74e+04)	8.64e+05 (2.03e+05)	1.04e+06 (2.73e+05)
$f_{s3,4}$	9.82e+04 (3.94e+04)	1.07e+06 (3.32e+04)	9.62e+05 (1.34e+05)	1.17e+06 (1.18e+05)
α	0.907 (0.00271)	0.901 (6.52e-04)	0.901 (8.82e-04)	0.904 (0.00177)

Similar to the results of patient 1 in Section 4.3.2 in the main text, the inferred flow and pressure waveforms of patients 2, 3 and 4 line up similarly to the observed data. Patient 4's predicted systolic and diastolic pressures (Figure 4b) are slightly misaligned, indicating biased parameter posterior distributions, potentially due to model mismatch or one of the invalid assumptions listed in Section 4.2.3 in the main text.

References

[1] Alyssa M. Taylor-LaPole, L. Mihaela Paun, Dan Lior, Justin D. Weigand, Charles Puelz, and Mette S. Olufsen. Parameter selection and optimization of a computational network model of blood flow in single-ventricle patients. *J. R. Soc. Interface*, 22(223):20240663, 2025.

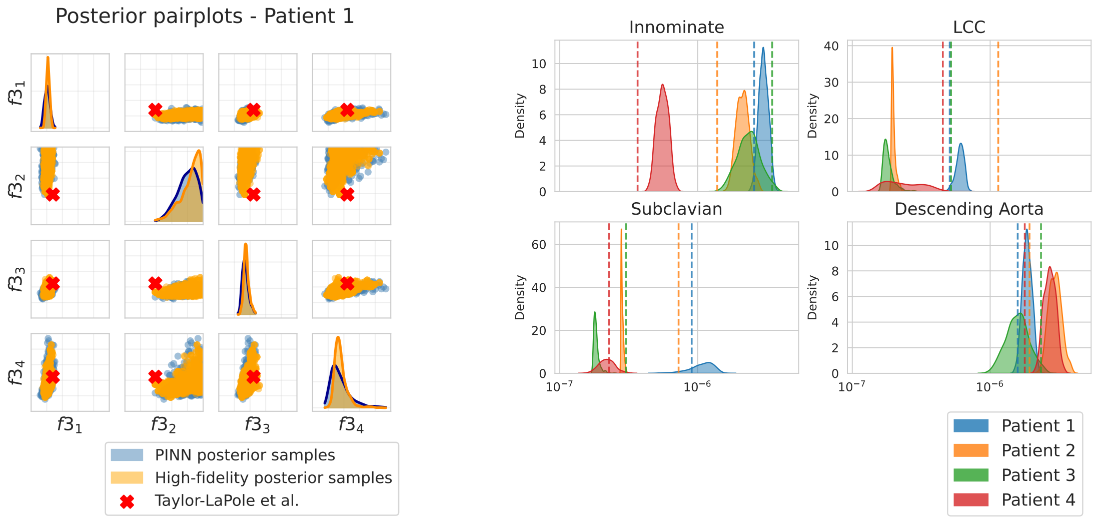

(a) (b)

Figure 1: (a) Pairplots between posterior samples of the stiffness parameters corresponding to the 4 vessel groups, obtained using both the PINN as an emulator and the PDE solver as the predictive model for Patient 1. Each of the figures is constrained to the parameters' ranges. Red crosses indicate the point estimates obtained by Taylor-LaPole et al. [1] in the original study. (b) Compliance distributions obtained via the parameters' posteriors in four arteries. The vertical lines indicate the compliances obtained in the original study. The compliances are roughly in the same range, with differences in some of the patients due to a different tapering strategy applied to the descending aorta.

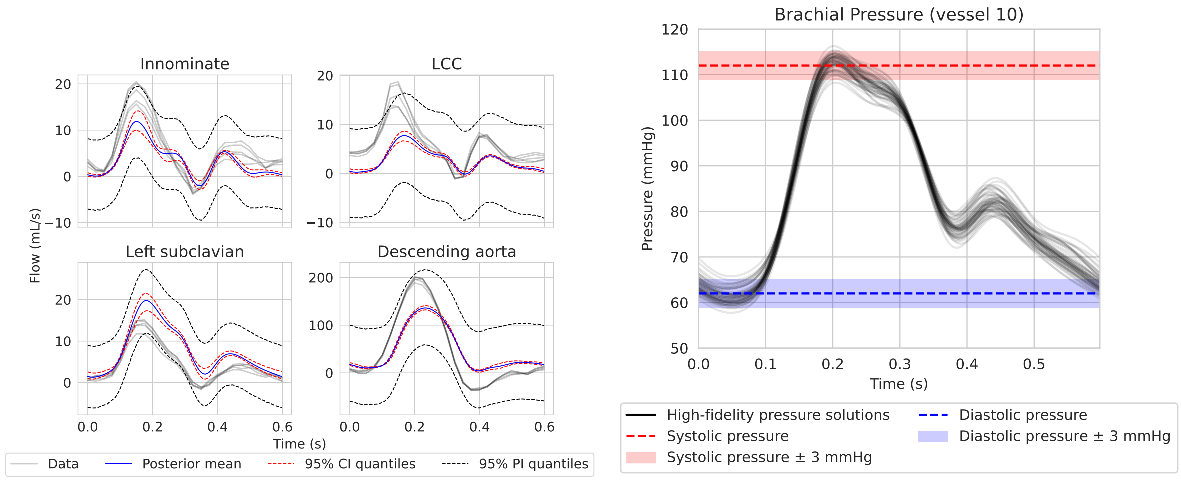

(a) (b)

Figure 2: Patient 2 inference results: (a) 95% posterior credible and prediction intervals in each of the 4 vessels used for inference. (b) 95% posterior credible intervals in the brachial artery.

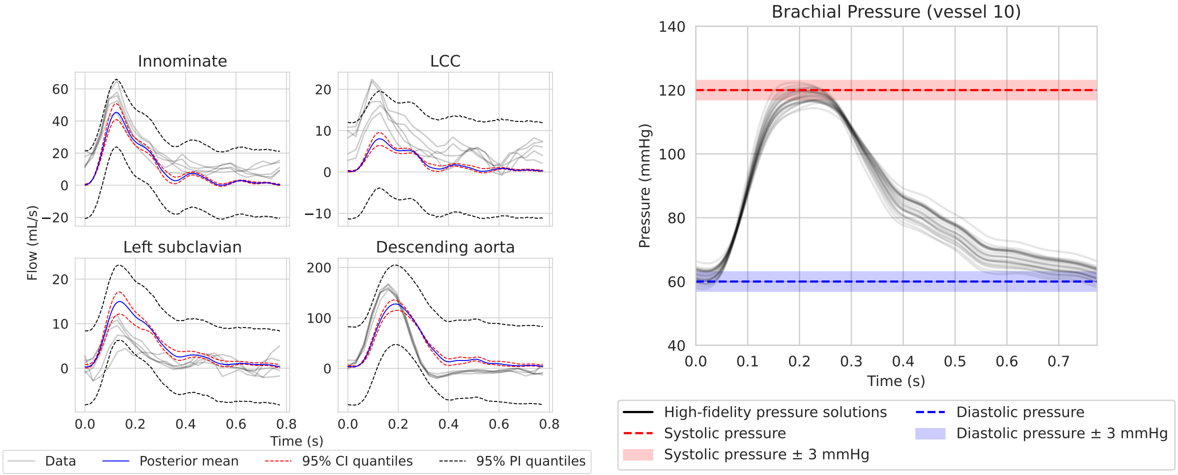

(a) (b)

Figure 3: Patient 3 inference results. (a) 95% posterior credible and prediction intervals in each of the 4 vessels used for inference. (b) 95% posterior credible intervals in the brachial artery.

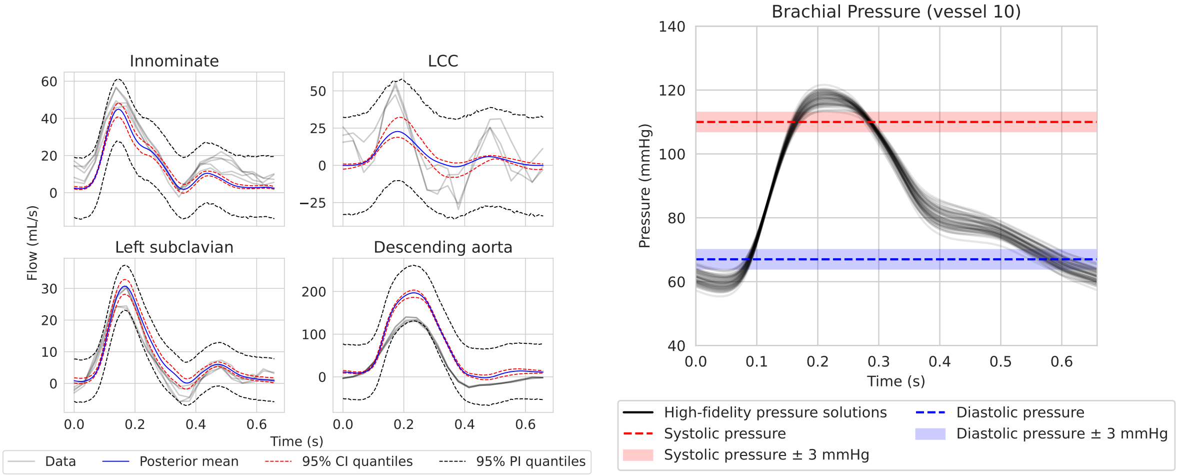

(a) (b)

Figure 4: Patient 4 inference results: (a) 95% posterior credible and prediction intervals in each of the 4 vessels used for inference. (b) 95% posterior credible intervals in the brachial artery.
